# Variation of the Fermi level and the electrostatic force of a metallic nanoparticle upon colliding with an electrode†
†Electronic supplementary information (ESI) available: Finite element model description, justification of model assumptions, schematic descriptions of the Fermi level changes upon NP collision with an electrode, the simulations of double layer capacitance of an electrode calculated with different double layer models, 3D plots corresponding to the contour plots of Fig. 1, the effect on the electrolyte concentration and NP radius on the potential distribution, surface charge density plots on the electrode and NP and their comparison with those calculated analytically in the absence of electrolyte, details of the calculations of the interaction between dissimilar parallel plates, and a further discussion of the approximate analytical expressions for the force between NP and electrode in the presence of electrolyte. See DOI: 10.1039/c7sc00848a


**DOI:** 10.1039/c7sc00848a

**Published:** 2017-05-09

**Authors:** Pekka Peljo, José A. Manzanares, Hubert H. Girault

**Affiliations:** a Laboratoire d’Electrochimie Physique et Analytique (LEPA) , École Polytechnique Fédérale de Lausanne (EPFL) , Rue de l’Industrie 17 , CH-1951 Sion , Switzerland . Email: pekka.peljo@epfl.ch; b Department of Thermodynamics , Faculty of Physics , University of Valencia , c/Dr. Moliner, 50 , E-46100 Burjasot , Spain

## Abstract

When a metallic nanoparticle (NP) comes in close contact with an electrode, its Fermi level equilibrates with that of the electrode if their separation is less than the cut-off distance for electron tunnelling. In the absence of chemical reactions in solution, the charge on the metallic nanoparticle is constant outside this range before or after the collision. However, the double layer capacitances of both the electrode and the NP are influenced by each other, varying as the function of distance. Because the charge on the nanoparticle is constant, the outer potential of the metallic NP and hence its Fermi level varies as the capacitance changes. This effect is more pronounced for small particles (<10 nm) in diluted supporting electrolyte solutions, especially if the metallic nanoparticle and the electrode have different potentials of zero charge. Nanoparticles were found to be more electrochemically active in the vicinity of the electrode. For example, the outer potential of a positively-polarized 2 nm radius NP was predicted to decrease by 35 mV or 100 mV (depending on the electrostatic model used to describe the electric double layer), when the NP moved from an electrode at 1 V (*vs.* its pzc) to the bulk. The force between the equilibrated NP and the electrode is always repulsive when they have the same pzc. Otherwise there can be an attraction even when the NP and the electrode carry charges of the same sign, due to the redistibution of surface charge density at both the NP and electrode surface.

## Abstract 2

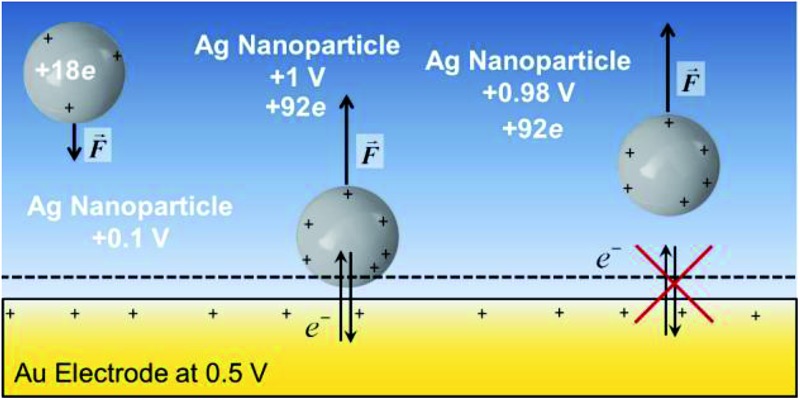
The nanoparticle potential varies with the distance from the electrode, and sometimes like attracts like.

## Introduction

Recently, nanoelectrochemistry has become a hot topic, as highlighted, for example, by the special issue in Accounts of Chemical Research.^1–6^ Much of the focus has been on observing the current response of the nanoparticle (NP) landing on a small electrode. As described in recent reviews, NPs can stick to the electrode, or collide and move back to the bulk. NPs can dissolve upon contact, or they can catalyse electrochemical reactions that are kinetically limited on the substrate electrode, like H_2_ or O_2_ evolution, H_2_O_2_ or O_2_ reduction, metal deposition, *etc*.^1–8^ In this work, classical electrostatic models are used to describe the difference in the Fermi levels of an electrode and single metallic NP immersed in an electrolyte solution as a function of their separation for different values of the NP radius and of the electrode potential. Generally, the Fermi level of the electrode is imposed by an external power supply. Charge transfer between the electrode and the NP is possible when their separation is lower than the cut-off distance for electron tunneling. Thus, when talking of NP collisions with the electrode, no physical contact is actually needed. The kinetics of electron tunneling could be described by, *e.g.*, the orthodox theory and the time-average value of the NP charge would then be evaluated. This kind of stochastic charge fluctuation has been considered for example for nanoscale bipolar electrodes.^9^ The average NP charge is a continuous variable which does not exhibit quantized charging effects when the thermal energy is larger or similar to the energy difference between consecutive charge states of the NP (see ESI†). The charge transferred to equilibrate the Fermi levels depends on the NP size, and hence on its capacitance.^10^ It is accepted that the NP can have charges of partial electrons as this charge is considered to be a time-average value.

An alternative approach is to consider the NPs as multivalent redox “molecules” with equally spaced, formal redox potentials. The condition of electrochemical equilibrium between an electrode and a solution of NPs and is equivalent to the Fermi level equilibration. The electrode potential then determines the relative populations of the different oxidation states, and hence the average oxidation state of the NPs in the solution. The average charge number of the NPs in the solution is a continuous variable analogous to the NP charge in our approach. The important point to be stressed is that the Fermi level of the electrons in the NP, analogous to the electrochemical potential of the electrons in a solution containing NPs, is a continuous variable that describes the tendency to exchange electrons in order to reach electrochemical equilibrium with the electrode. The Fermi level of the electrons in the NP is not necessarily equal to the energy of the NP in one of its oxidation states. In a solution of NPs, the transfer of just a single electron from the electrode to one NP in oxidized state *z* causes a dramatic change in the energy of this NP, but the electrochemical potential of the electrons in the solution does not undergo any dramatic change. The latter is a property of the solution that is equivalent to the electrochemical potential of the electrons in the single NP that we consider in this work.

Any change in the separation distance between the NP and the electrode affects also the capacitance of the NP, driving charge transfer between the two objects to adjust their Fermi levels. This equilibration can take place only when the NP is close enough for the electron tunnelling to occur. The charge of the particle will continue to change until the cut-off distance is reached. After this point, the charge is constant but the outer potential of the NP will change in response to the change in the capacitance. This is clear in vacuum or in air, but the purpose of this article is to show that this effect is also significant in electrolyte solutions, where the electrostatic interactions are screened by the electric double layer. With low supporting electrolyte concentrations typically used in NP impact experiments to avoid aggregation, the Debye length can be several nm, while the cut-off distance for electron tunnelling is shorter (<1.5 nm).^11^ Hence, the capacitance of a NP will be higher close to the electrode surface. When the NP moves away from the surface, the Fermi level of the electrons will actually vary even if the NP charge remains constant! Negatively charged NPs will have less negative outer potential (Fermi level decreases), while the Fermi level will increase for positively charged NPs!

Recently, there has been a discussion in the literature whether the dissolution of NPs takes place in one or multiple steps.^7,12–18^ For example, Unwin *et al.* ^16^ have shown experimentally for the first time that large NPs partially dissolve in multiple collision events, and this observation was confirmed by Long *et al.* ^17^ and White and Zhang *et al.* ^18^ However, the exact mechanism of the NP collisions and subsequent leaching has not been clarified. For example, these large NPs seem to be consumed in a series of “bites”, with the NP dissolving closest to the electrode.^16^ However, it is not clear why the NP dissolution does not take place at the outer surface, as the dissolution would be most likely controlled by the mass transfer of dissolved ions away from the NP surface. If this is the case, dissolution events should be terminated by NP diffusion, and more likely by electrostatic interactions, as proposed recently by White and Zhang *et al.* ^18^ However, the effect of contact electrification was neglected, and the electrostatic effects were not quantified. Further work is required to resolve the exact mechanism.

As stated above, the NP will equilibrate its Femi level with that of the electrode, but this does not mean that the outer potentials will be equal. For example, Ag and Au have different potentials of zero charge of –0.44 V and 0.18 V *vs.* SHE,^19,20^ so that at electrode potentials of 0.6 V *vs.* Ag/AgCl, typically used for Ag dissolution on a gold electrode, the outer potential of Au will be *ca.* 0.6 V while the outer potential of Ag will be *ca.* +1.2 V. This difference in outer potentials is due to the contact electrification (when two electrically-neutral metals are connected, electrons from the metal with the lower work function will flow into the metal with the higher work function, resulting in a Volta potential difference),^21,22^ modified by solvent–metal interactions and other surface modifications when the metal is brought into contact with the solvent. Trasatti and Lust have comprehensively reviewed this topic.^19^ Of course, the additional effects of ligands such as citrate and of the electric double layer affect the apparent potential of zero charge (pzc) of the NP.

## Theory

### Poisson–Boltzmann equation

An electric double layer forms around any charged object in an electrolyte solution, where this surface charge affects the solvent molecules and the ions in the solution.^23^ The Poisson equation

1 *ε*_0_*ε*_r_∇^2^*ψ* = –*ρ*

relates the electrostatic potential *ψ* to the local space charge density2
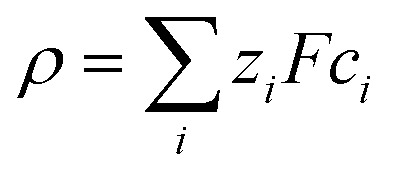
where *ε*_0_ and *ε*_r_ are the permittivity of vacuum and the relative permittivity. The concentrations of ions follow the Boltzmann distribution3
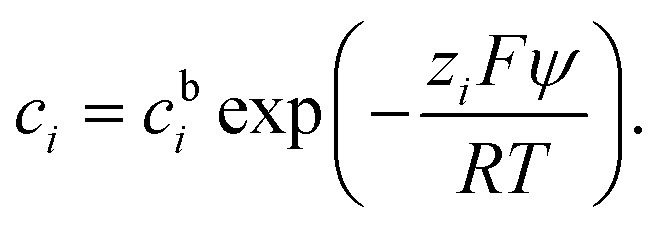



In a binary symmetric electrolyte (*z*_+_ = |*z*_–_| = *z*) the Poisson–Boltzmann equation (PBE) is

4 ∇^2^*φ* = *κ*^2^ sinh *φ*

where *φ* ≡ *zFψ*/*RT* is the dimensionless potential, *κ* = (2*c* ^b^ *z* ^2^ *F* ^2^/*ε*_0_ *ε*_r_ *RT*)^1/2^ is the Debye parameter (or reciprocal Debye length), and *c*b+ = *c*b– = *c* ^b^ is the ionic concentration in the bulk solution (where *ψ* = 0).^23^

The Stern modification adds inner and outer Helmholtz planes next to the charged surfaces. The outer Helmholtz layer is the closest approach of solvated ions to the surface, while the inner Helmholtz layer consists of mostly organized solvent dipoles and may also contain some specifically adsorbed ions that have lost their solvation shell. In the absence of specific ion adsorption, the potential satisfies the Laplace equation ∇^2^ *ψ* = 0 within the Stern layer.^23^

### Spherical geometry. Capacitance of the isolated NP

The PBE in spherical geometry, *φ*′′ + (2/*r*)*φ*′ = *κ* ^2^ sinh *φ*, lacks a general analytical solution (see ESI†). When the NP is considered as a charged conducting sphere of radius *R*_NP_ and the term (2/*r*)*φ*′ is approximated by –(4*κ*/*R*_NP_)sinh(*φ*/2),^24^ the first integration of the PBE yields the relation between the surface charge density and the surface potential *φ*_NP_ = *φ*(*R*_NP_)5
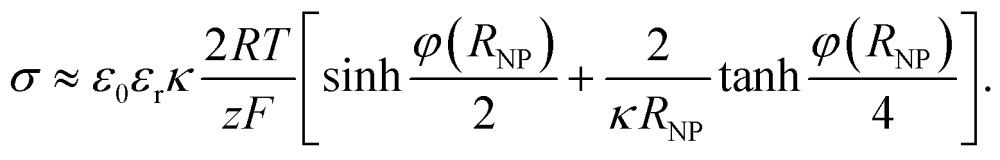
Hence, the differential capacitance of the isolated NP is6
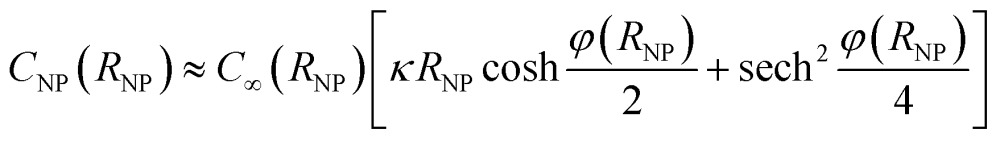
where *C*_∞_(*R*_NP_) = 4π*ε*_0_ *ε*_r_ *R*_NP_ is its value in absence of electrolyte. When *κR*_NP_ ≫ 1, the areal capacitance *C*_NP_/4π*R*_NP_^2^ reduces to that of Gouy–Chapman, *C*_GC_ = *ε*_0_ *ε*_r_ *κ* cosh(*φ*_E_/2) where *φ*_E_ is the potential at the charged surface.

Including an uncharged Stern layer of thickness *δ* free of ions, the relation between the potential values at the boundaries of this layer is7
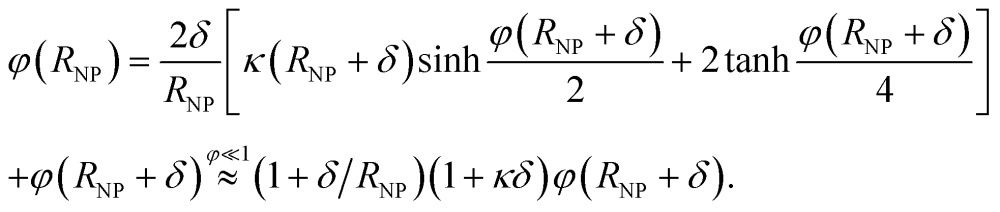



The NP capacitance is then obtained from 1/*C*_NPS_ = 1/*C*_*δ*_ + 1/*C*_NP_(*R*_NP_ + *δ*) where *C*_*δ*_ = *C*_∞_(1 + *R*_NP_/*δ*) corresponds to a spherical capacitor of inner radius *R*_NP_ and outer radius *R*_NP_ + *δ*, and *C*_NP_(*R*_NP_ + *δ*) is the capacitance of a NP of radius *R*_NP_ + *δ* (*i.e.*, eqn (6) with *R*_NP_ + *δ* replacing *R*_NP_).

The capacitance of weakly-charged NPs (*φ*_NP_ ≪ 1) simplifies to *C* *φ*≪1NPS = *C*_∞_[1 + *κR*_NP_/(1 + *κδ*)]. For a NP of radius *R*_NP_ = 2 nm in a 1 : 1 aqueous electrolyte of *c* ^b^ = 10 mol m^–3^ (1/*κ* = 3.03 nm, *ε*_r_ = 78) with a surface potential *RTφ*(*R*_NP_)/*zF* = 50 mV, and a potential *RTφ*(*R*_NP_ + *δ*)/*zF* = 39 mV at the outer boundary of the Stern layer of thickness *δ* = 0.33 nm, these capacitances are *C*_NP_(*R*_NP_ + *δ*) = 37.7 aF and *C*_NPS_ = 28.8 aF, and the areal value is *C*_NPS_/4π*R*_NP_^2^ = 0.57 F m^–2^. For comparison, the areal capacitance of an electrode (with a Stern layer) at *φ*_E_ *RT*/*zF* = 50 mV is *C*_E_ = [1/*C*_GC_ + *δ*/*ε*_0_ *ε*_r_]^–1^ = 0.28 F m^–2^, where *C*_GC_ ≈ *ε*_0_ *ε*_r_ *κ* cosh[*φ*_E_/2(1 + *κδ*)] = 0.32 F m^–2^ is the contribution from the electrolyte solution.

### Force on the NP

Once the potential distribution is known, the electrostatic force ***F*** on the NP is evaluated as8
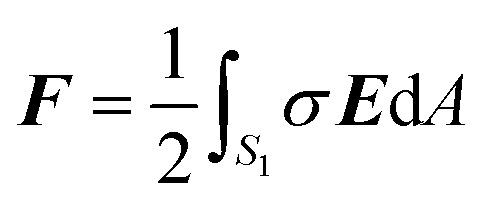
where ***E*** is the electric field at the NP surface, *σ* is its surface charge density, and *S*_1_ is the surface just outside the NP.^25^

### Interaction between charged parallel plates

An outline of the interaction between plates helps to understand the numerical results for the interaction between a spherical NP and an electrode. The potential distribution at a distance *z* from a plate with surface potential *φ*_NP_ > 0 is

9 *φ*(*z*) = 4artanh[tanh(*φ*_NP_/4)exp(–*κz*)].

The electrostatic contribution to the force density on the plate10
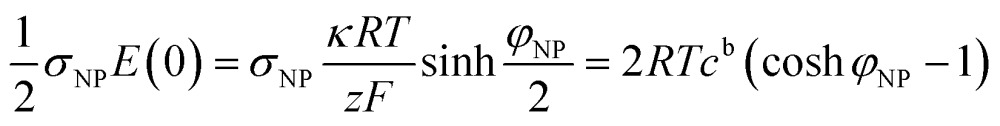
is directed towards the solution. The charge density *σ*_NP_ = *ε*_0_ *ε*_r_ *E*(0) on the plate affects the ionic distribution. The total ion concentration close to the plate is *c*_+_(0) + *c*_–_(0) = 2*c* ^b^ cosh *φ*_NP_, larger than the bulk value 2*c* ^b^. The osmotic pressure difference between the plate surface and the bulk, Δ*Π* = 2*RTc* ^b^(cosh *φ*_NP_ – 1), exerts a force on the plate that compensates the electrostatic one, eqn (10), as it has the same magnitude and opposite direction.

If we place a second metal plate at a distance *s* from the plate at *z* = 0 their interaction can be attractive, repulsive or null depending on its potential *φ*_E_. If *φ*_E_ is equal to *φ*(*s*) given by eqn (9), the charge density on the plate at *z* = *s* is11
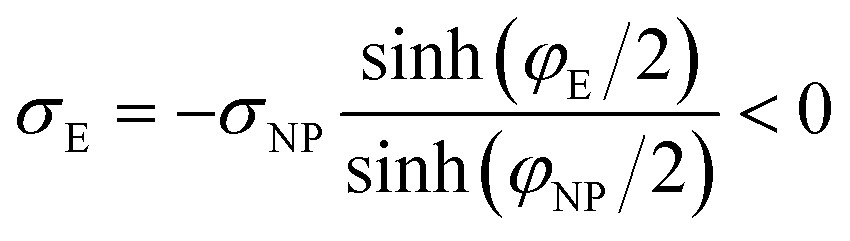
and there is no interaction, even though the plates have charges of opposite sign, because the effect on the plate at *z* = 0 due to the plate at *z* = *s* is the same as that due to the electrical double layer beyond *s*, 
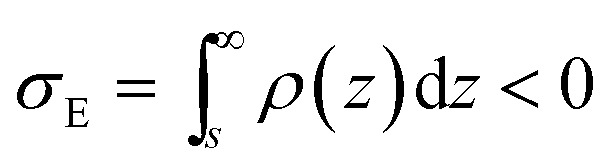
.

If *φ*_E_ satisfies *φ*_E_ > *φ*(*s*) > 0, the charge density on the plate at *z* = *s* is more positive than *σ*_E_ in eqn (11) and the interaction between the plates is repulsive, even though *σ*_NP_ > 0 > *σ*_E_. On the contrary, if *φ*_E_ satisfies *φ*(*s*) > *φ*_E_ > 0, its charge density is more negative than *σ*_E_ in eqn (11) and the interaction is attractive, even though *ψ*_NP_ > *ψ*_E_ > 0.

The capacitance matrix formalism of electrostatics^21^ can be used for conductors in electrolyte solutions provided that the potentials are small and the PBE can be linearized. When the plates interact at constant charge, their surface potentials decrease with increasing separation *s*.^26^ If *ψ*_E∞_ = *σ*_E_/*C*_GC∞_ and *ψ*_NP∞_ = *σ*_NP_/*C*_GC∞_ > *ψ*_E∞_ > 0 are the potentials at large *s*, with *C*_GC∞_ = *ε*_0_ *ε*_r_ *κ*, then the values at finite separation are12
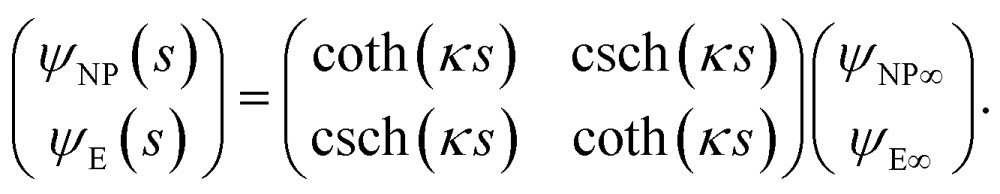
When the plates interact at constant potential, their charge densities vary with *s* as13
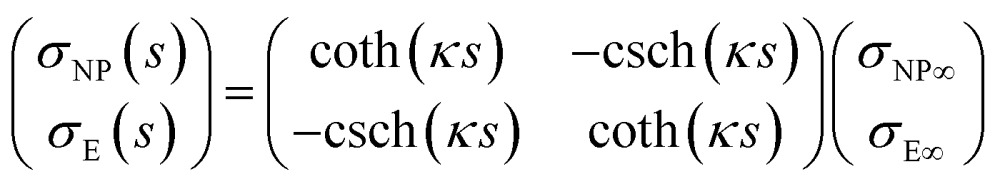
where *σ*_E∞_ = *C*_GC∞_ *ψ*_E_ > 0 and *σ*_NP∞_ = *C*_GC∞_ *ψ*_NP_ > *σ*_E∞_ > 0 are the values at large separations. Eqn (13) implies that the capacitance of a plate is a decreasing function of *s*, *C*_NP_(*s*) = (∂*σ*_NP_/∂*ψ*_NP_)_*s*,*ψ*_E__ = *ε*_0_ *ε*_r_ *κ* coth(*κs*). The charge density *σ*_NP_ on the plate with higher potential remains positive. However, *σ*_E_(*s*) becomes negative if *κs* < arcosh(*ψ*_NP_/*ψ*_E_). At even shorter distances, *κs* < ln(*ψ*_NP_/*ψ*_E_), the normal stress14
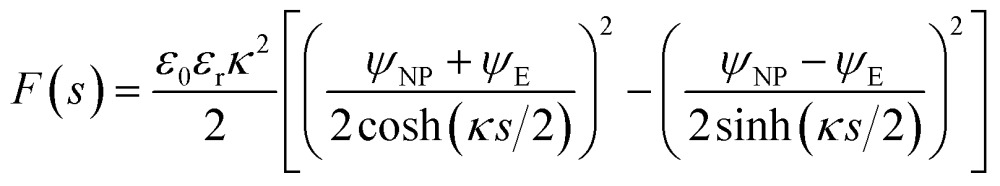
is negative (see ESI†), corresponding to an attraction between plates with *ψ*_NP_ > *ψ*_E_ > 0 and charges densities of opposite signs.^27^ Repulsion dominates when increasing *ψ*_NP_ and *ψ*_E_ at constant *ψ*_NP_ – *ψ*_E_.

### Interaction between NP and electrode in electrolyte solution

The interaction of two spheres in the absence of an electrolyte solution has been described in ref. 21, and the interaction of the sphere and a plate in the absence of an electrolyte is described in the ESI.† For large spherical NPs (*κR*_NP_ ≫ 1) and low potentials (*φ* ≪ 1), the potential energy when the NP and a planar electrode in electrolyte solution have constant potentials at a separation *s* is^26,28^
15
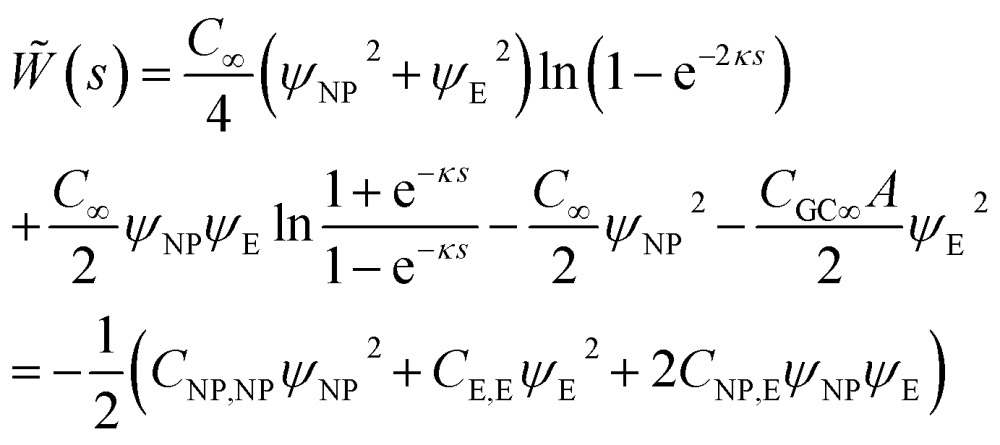
where in the last step we have introduced the capacitance matrix coefficients. The capacitance of the NP16
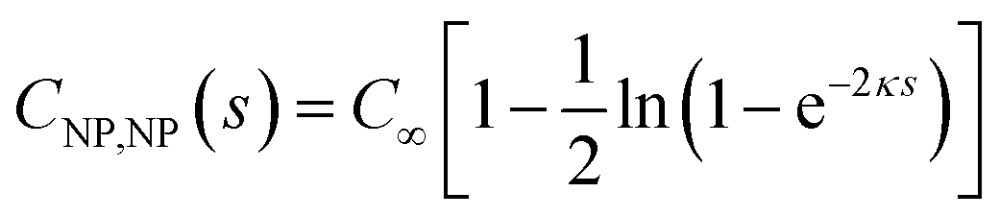
is a decreasing function of *s* and differs from *C*_∞_ by less than 1% for *s* > 2/*κ*. Similarly to the case of parallel plates, the charge on the spherical NP varies with *s*; in particular, when *ψ*_NP_ = *ψ*_E_ the charge *Q*_NP_ = *C*_∞_ *ψ*_NP_[1 – ln(1 + e^–*κs*^)] decreases as it approaches the electrode (at constant potentials), but it may also increase with decreasing *s* when *ψ*_NP_ > *ψ*_E_ > 0.

### Modifications of the Poisson–Boltzmann equation

A well-known limitation of the PBE is that the ions are considered as point charges, resulting in abnormally high surface concentrations at high polarisations. Cervera *et al.* took into account the steric effects using a modified Boltzmann distribution, which replaces *c*b*i* in eqn (3) by *c*b*i*/[1 + 2*v*_*i*_ sinh^2^(*φ*/2)], where *v*_*i*_ = 2*N*_A_ *a*_*i*_^3^ *c*b*i* is a packing parameter, *N*_A_ is Avogadro's constant, and *a*_*i*_ is the diameter of the solvated ion *i*.^29–32^ Thus, with increasing polarisation, the surface concentration of counterions cannot exceed the steric limit 1/(*N*_A_ *a*_*i*_^3^). Similar expressions were proposed in the 1940s and 1950s by a number of authors, as reviewed by Bazant.^33^ The modified PBE becomes then17
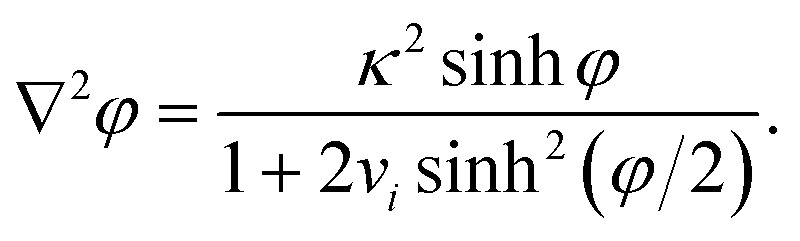



Additionally, the relative permittivity of the solution can be modified by the electric field, as described for example by the Booth model:^34–37^
18
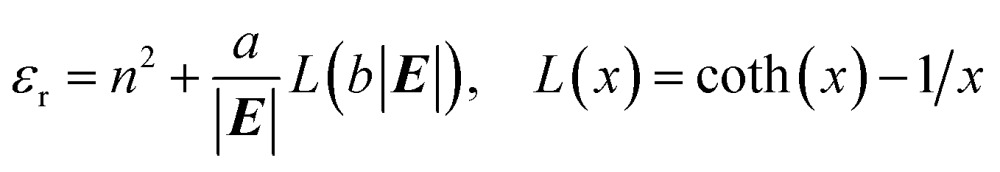

19
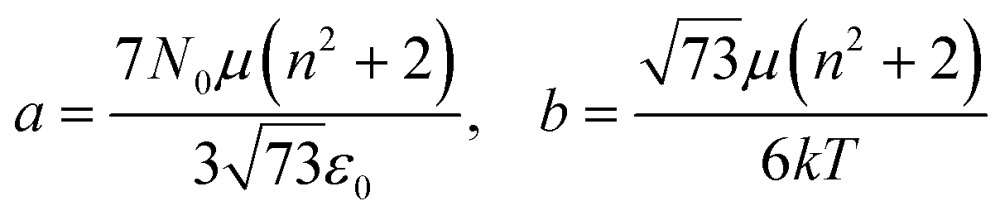
where *n* is the optical refractive index, |***E***| is the electric field strenght, *N*_0_ = 3.343 × 10^28^ m^–3^ is the number density of molecules, *μ* is the water molecule dipole moment, and *k* is Boltzmann's constant. The dipole moment of water was adjusted to *μ* = 6.6621 × 10^–30^ C m to obtain *ε*_r_ = *n* ^2^ + *ab*/3 = 78 in weak fields |***E***| < 1 MV m^–1^.^37^ The potential distribution is obtained from the numerical solution of these equations. Although the finite ion size effects are take into account, the validity of continuum models for the description of the double layer around NPs of 1 nm radius might be questionable. The modified PBE flattens out the oscillations in the space charge density due to the finite size effects of the electrolyte ions, but the overall surface charge on the NP is not expected to be significantly influenced.^21^ Simulations of the electrode and NP capacitances in electrolyte solution calculated with different models are shown in the ESI.†

## Computational methods

**Scheme 1:**
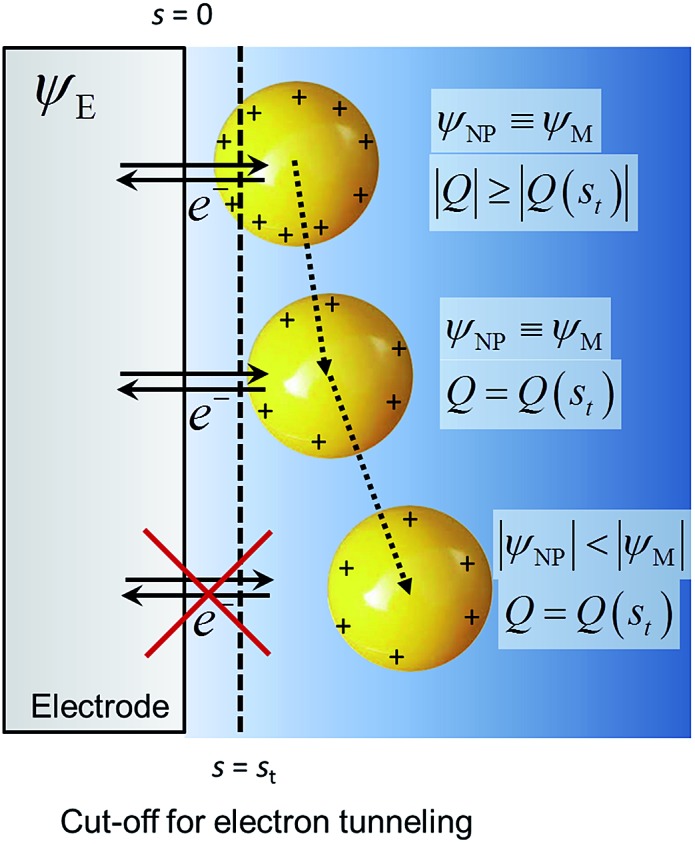
Nanoparticle potential and charge after collision with the electrode. *ψ*_E_ = electrode potential, *ψ*_NP_ = nanoparticle potential, *Q* = charge and *s* = separation from the electrode surface.

The system shown in Scheme 1 was studied by finite element simulations with COMSOL Multiphysics 5.2a, as described in the ESI.† The NP was simulated as a conducting sphere. All the charged metal surfaces were considered to have a Stern layer of the radius of the hydrated K^+^ ion (0.33 nm)^38^ free of supporting electrolyte, and the modified PBE was used to calculate the distributions of potential and concentrations of the supporting electrolyte species. The relative permittivity of the solution was considered to be either constant and equal to that of pure water (model I) or dependent on the electric field intensity as described by the Booth model [model II, eqn (18) and (19)].

All potentials are given with respect to the potential of zero charge (pzc) of the electrode. Thus, for instance, the pzc of the NP is denoted by *E*_pzc_ and a value *E*_pzc_ = 0 indicates that the NP and the electrode have the same pzc. The electrode and the NP were considered to have the same Fermi level

20 *ψ*_NP_ + *E*_pzc_ = *ψ*_E_

when their separation was lower than the cut-off distance for electron tunnelling. For example, a NP with *E*_pzc_ = –0.5 V equilibrated with an electrode at its pzc (*ψ*_E_ = 0) has a potential *ψ*_NP_ = 0.5 V. If the NP moves further away, its charge is constant and equal to the one it had at the cut-off distance for the electron tunnelling. This cut-off distance was arbitrarily chosen as *s*_t_ = 1 nm; alternatively, it could be calculated, for example, by the Simmons' model.^11,39^

The capacitance of a NP in the vicinity of an electrode differs from that of an isolated NP discussed above because the potential distribution around the NP is affected by the electrode. The differential capacitance *C*_NP_ = ∂*Q*_NP_/∂*ψ*_NP_ of the NP was calculated as the function of both the separation distance *s* and *ψ*_E_. For each value of *ψ*_E_, the NP was first placed at the cut-off distance of electron tunnelling (*s*_t_ = 1 nm) and was equilibrated with the electrode to obtain the NP charge; its potential *ψ*_NP_(*s*_t_) = *ψ*_E_ – *E*_pzc_ was given by eqn (20). For larger NP-electrode separation, *s* ≥ *s*_t_, and the same *ψ*_E_, the NP potential *ψ*_NP_ and the NP differential capacitance *C*_NP_ were unknown. The NP potential *ψ*_NP_ was varied from *ψ*_NP_(*s*_t_) to slightly lower values and the NP charge *Q*_NP_(*ψ*_NP_,*s*,*ψ*_E_) was calculated for every value of *ψ*_NP_. From these values, *C*_NP_(*s*,*ψ*_E_) = (∂*Q*_NP_/∂*ψ*_NP_)_*s*,*ψ*_E__ was evaluated and then, the actual value of *ψ*_NP_(*s*) was estimated from the known NP charge, considered to be constant after the NP-electrode collision, that is, *Q*_NP_(*ψ*_NP_(*s*),*s*,*ψ*_E_) = *Q*_NP_(*ψ*_NP_(*s*_t_),*s*_t_,*ψ*_E_).

The differential capacitance of the electrode was firstly evaluated for a planar surface in 1D geometry, considering the different models for the double layer, and a good agreement with the analytic and numerical results was obtained (see ESI†). Then, the capacitance of the electrode in 2D axis symmetrical geometry was evaluated, showing a good agreement with the 1D simulations.

The Fermi level of the electrons in the NP (and, hence its pzc) varies with the NP size, as shown in a recent review.^40^ Additionally, polycrystalline electrode materials have patches with different pzc values,^21^ which may also affect the exchange of electrons with the NP. For simplicity, these effects are not considered in this paper.

## Results and discussion

### Potential and capacitance of the NP (same pzc as electrode)

**Fig. 1:**
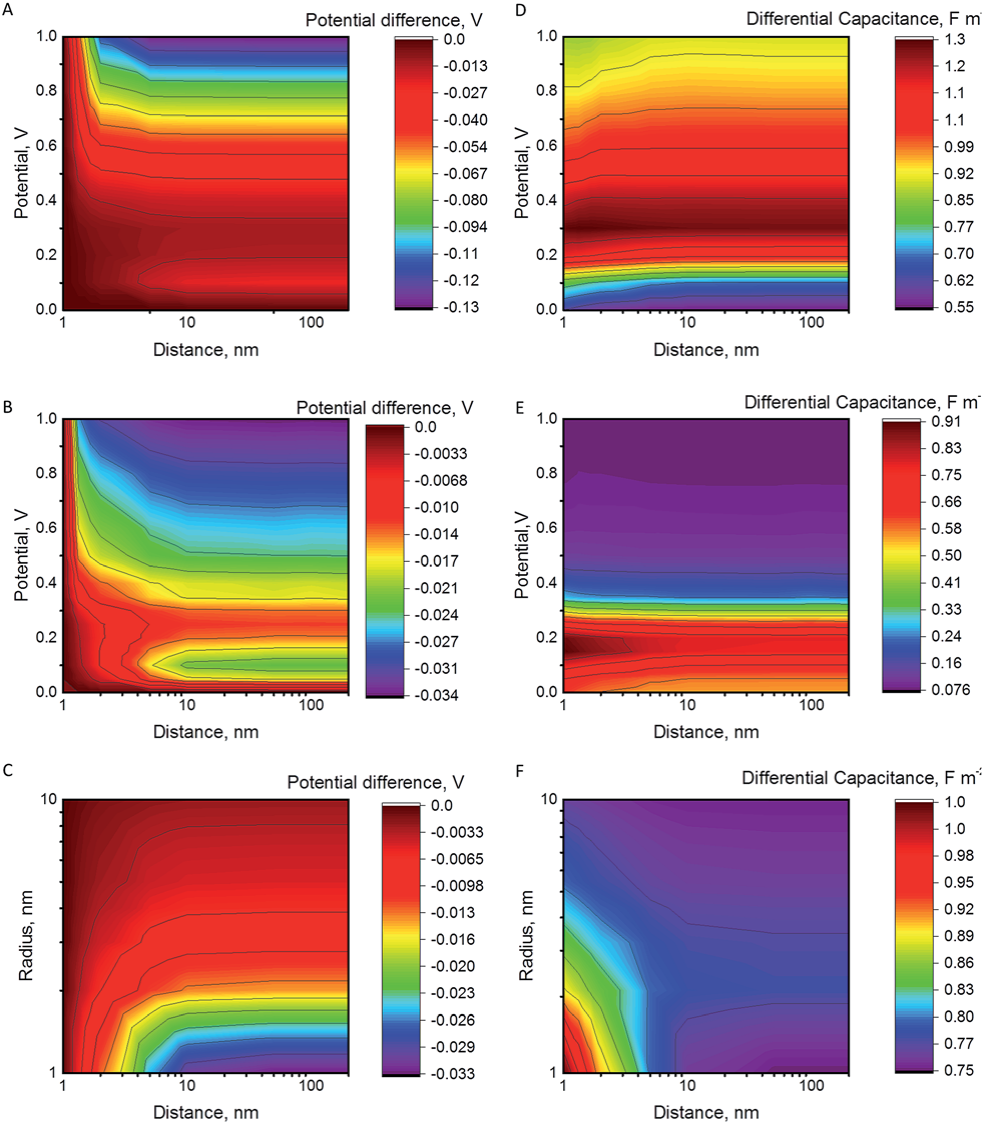
Potential difference between the NP and the electrode after collision as a function of their separation and of the electrode potential for *R*_NP_ = 2 nm, calculated: (A) with *ε*_r_ = 78 (model I) and (B) with the Booth model for the relative permittivity (model II). Differential capacitance as a function of NP electrode separation and NP potential for *R*_NP_ = 2 nm and *E*_pzc_ = 0 V, calculated: (D) with model I and (E) with model II. Effect of the NP radius on: (C) the potential difference and (F) the differential capacitance, calculated with model II and electrode potential of 0.2 V.

The potential difference between the NP and the electrode after a collision as a function of the increasing separation distance, the electrode potential and the NP radius are shown in Fig. 1A–C. The differential capacitance *C*_NP_ = ∂*Q*_NP_/∂*ψ*_NP_ is shown in Fig. 1D–F (for 3D plots see Fig. S3 in the ESI†). In the case of constant *ε*_r_ (model I), the differential capacitance of the electrode increases as a function of potential until no more ions can be packed at the electrode surface, followed by a slow decrease. The comparison with the results of model II, where the relative permittivity varies with the electric field strength, highlights that the capacitance of the NP has a significant effect on the observed behaviour. In model II, the capacitance decreases faster as a function of the increasing potential. Both models produce the famous camel-like dependence of differential capacitance on surface potential,^41^ but the decrease of capacitance at higher potentials is less pronounced with constant *ε*_r_. If the capacitance does not significantly vary as the function of the distance from the electrode, there is no change in the NP potential and hence no significant effect on the Fermi level of the electrons in the NP. However, if the capacitance varies significantly, the NP will become less reactive in the bulk. For example, model II predicts a decrease of 20 mV to 35 mV in the NP potential for *R*_NP_ = 2 nm, while model I results in higher potential decrease of 130 mV at 1 V.

Fig. 1 shows that NP radius is another important factor. For *R*_NP_ = 10 nm, model II predicts that the potential changes only 3 mV at 0.2 V, in comparison with 35 mV for *R*_NP_ = 1 nm, and for *R*_NP_ = 20 nm the potential decreases by 1.1 mV. If the potential of the electrode is increased to 1 V, the potential decrease for the NP of *R*_NP_ = 20 nm when moving into the bulk is 14 mV, compared to 34 mV for *R*_NP_ = 2 nm. Further increase of the NP radius to 50 or to 100 nm results in a potential decrease of 7 mV and 3 mV, respectively (electrode at 1 V). Hence, slurry electrodes utilizing micrometer sized particles show hardly any effect from the change of the capacitance. This is because the double layer of the electrode perturbs only a small part of the double layer of the large NPs, while this perturbation is larger for small NPs (see Fig. S4 in ESI† to see the electric potential for 2 and 10 nm NPs). If the supporting electrolyte concentration is increased, the thickness of the diffuse double layer decreases, and the effects will be smaller, as shown in Fig. S4C.†

The NPs may contact the electrode and diffuse away. If the NP collision is followed by an electrochemical reaction in the solution, (for example in a system where the electrode is electrocatalytically inert for a given redox couple, while the NP is active), the Fermi level of the NP will equilibrate with the Fermi level of the redox couple in the solution. Interestingly, the NP is most electrochemically reactive (it has more oxidative potential if positively charged, and more reductive potential if negatively charged) close to the electrode surface. While the NP is still close to the surface, the redox reaction with the redox couple in the solution will take place, perturbing the concentration ratio of the redox couple at the NP surface. As the particle moves further from the electrode surface, its Fermi level will change, and the redox reaction can proceed to the opposite direction as a response for this change. Of course, it should be considered whether the process is controlled by kinetics or by mass transfer.

### Potential and capacitance of the NP (different pzc values)

**Fig. 2:**
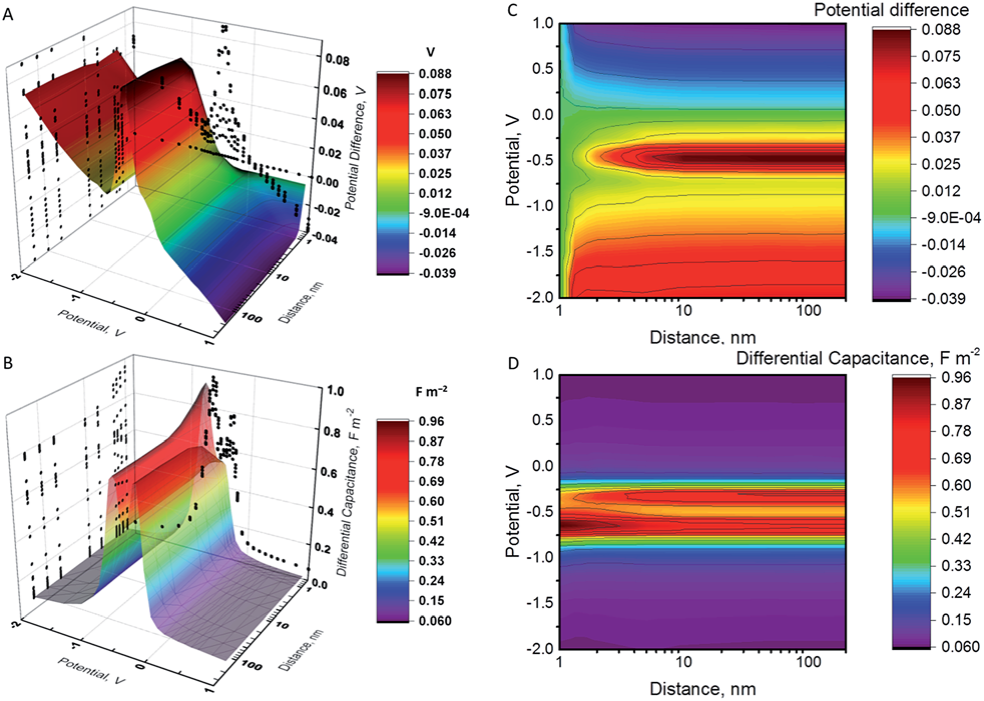
(A) and (C) Potential difference between the NP and the electrode after collision. (B) and (D) Differential capacitance of the NP. All results have been calculated using model II for *R*_NP_ = 2 nm and *E*_pzc_ = –0.5 V, and are presented as a function of the NP-electrode separation and of the electrode potential.

When a NP and an electrode with different pzc values, and hence different (real) chemical potentials of the electrons, get in contact, their Fermi levels equilibrate through contact electrification. A charge transfer takes place from the material with a lower work function into the material with a higher work function. This leads to a situation where the two different materials have the same Fermi level but different electrostatic (or outer) potentials. The changes in the differential capacitance of the NP can then be more drastic, as shown in Fig. 2.

In Fig. 2, we have considered a value *E*_pzc_ = –0.5 V which is actually close to the situation of AgNPs with a glassy carbon or a Au electrode, as Ag has a pzc of *ca.* –0.7 V *vs.* GC and –0.6 V *vs.* Au.^19,20,42^ The largest shifts in potential when moving the NP from the vicinity of the electrode into the bulk are observed close to the pzc of the NP. Interestingly, the sign of the change in the potential of the NP changes at electrode potentials slightly above 0 V. Below these potentials, the NP potential increases when it moves to the bulk, with the maximum of *ca*. +90 mV close to the pzc of the NP. When *ψ*_E_ > 0, *ψ*_NP_ – *ψ*_E_ is negative and increases in magnitude when the NP separates from the electrode. The differential capacitance curve shows an asymmetric shape close to the electrode, with the maximum on the negative side of the pzc value, while the symmetry is recovered in the bulk.

**Scheme 2:**
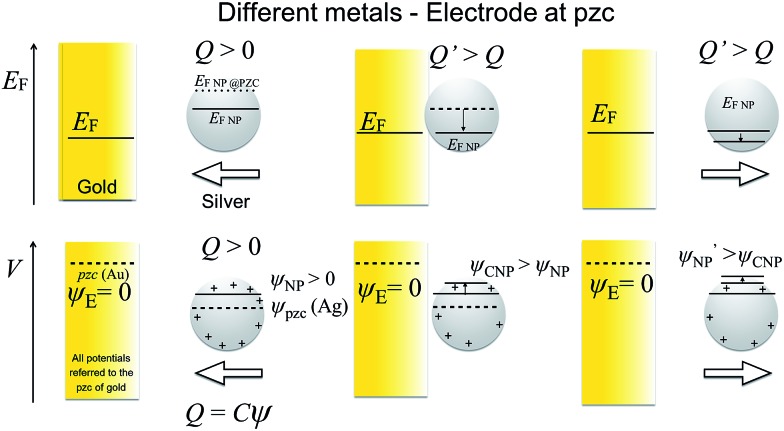
Electrode and NP made of different metals and the NP has *E*_pzc_ = –0.5 V. Top panel: Fermi levels. Bottom panel: Potential difference between metal and solution. The electrode is at its pzc. Before collision, the AgNP is positively charged (*ψ*_NP_ > 0) and, therefore has a lower Fermi level than at its pzc (*E*_F,NP_ – *E*_F,NP@pzc_ = –*eψ*_NP_ < 0). As the NP capacitance varies with the distance to the electrode, the potential difference between the NP and the solution varies both when the NP approaches the electrode and when departs from it after collision.

Additionally, the capacitance of the electrode changes when the NP moves farther away from its surface. However, this effect is significant only with very small electrodes, and this double layer perturbation is compensated by the change in the electrode capacitance during the approach, although the magnitude of the change depends on the NP potential when it approaches the electrode. Generally, the baseline of the measured current response in impact experiments shows some variations. This paper suggests that some of these variations could be ascribed to the changes in the capacitance of the electrode due to the NPs perturbing the electric double layer of the electrode, but careful comparison of the experiments with and without NPs would be required. The behaviour of NPs approaching and colliding with the electrode is summarized in the Scheme 2, as well as in Schemes S1 and S2† for the cases where the metals have the same pzc.

### Electrostatic force on the NP

**Fig. 3:**
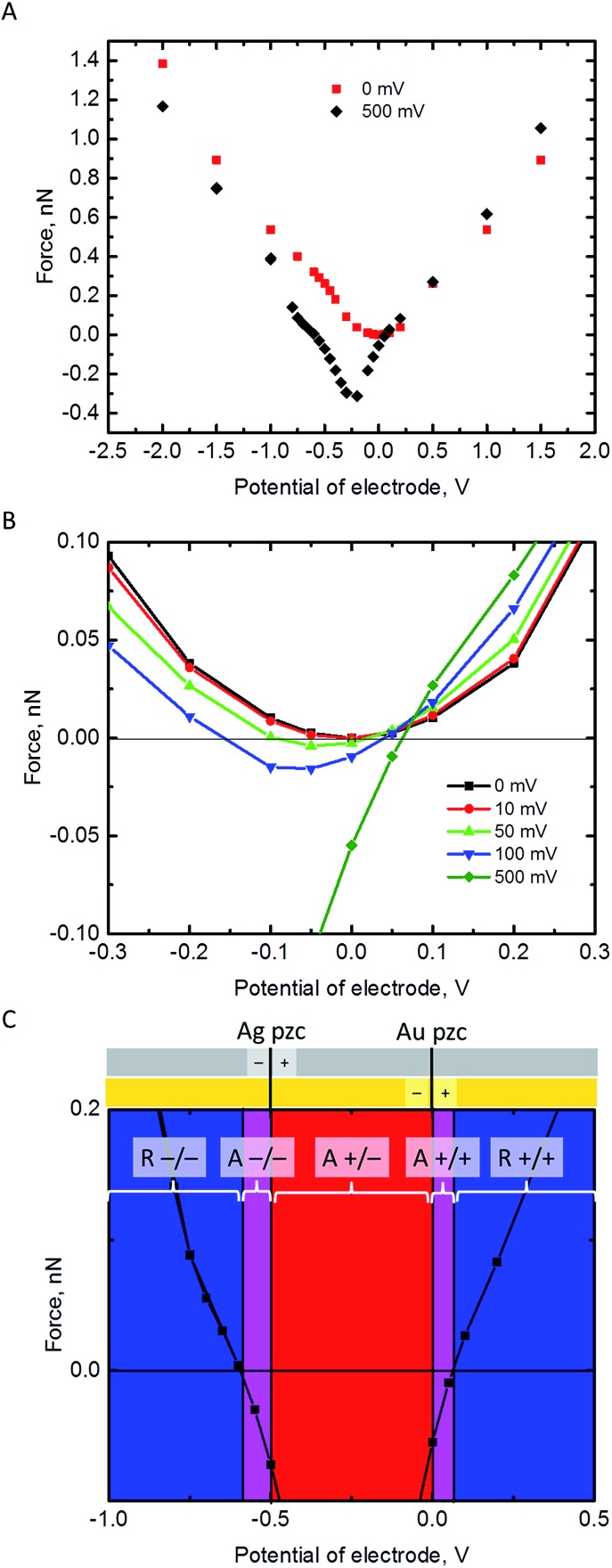
Electrostatic force on a 2 nm radius NP at a separation *s* = 1 nm from a Au electrode as a function of the electrode potential. The Fermi levels of NP and electrode are equilibrated, *ψ*_NP_ + *E*_pzc_ = *ψ*_E_, so that the NP potential is higher or equal to that of the Au electrode, *ψ*_NP_ – *ψ*_E_ = –*E*_pzc_ ≥ 0. (A) The cases of AuNP (–*E*_pzc_ = 0 mV) and AgNP (–*E*_pzc_ = 500 mV) considered over a wide range of electrode potentials. (B) Effect of the pzc difference on the force over a narrow range of electrode potentials around its pzc. (C) Regions of repulsion (R –/– and R +/+, blue) and attraction (A +/–, red) based on the pzcs of AgNP (*E*_pzc_ = –500 mV) and Au electrode, and the extended region of attraction due to the distribution of charge on the NP and electrode (A –/– and A +/+, violet).

The electrostatic force on the NP has been calculated at a separation of 1 nm from the Au electrode for different values of its pzc (Fig. 3A and B). In principle, repulsion when NP and electrode have potentials of the same sign, and attraction when they have different sign, could be expected. However, the discussion on the interaction between dissimilar parallel plates in the Theory section has evidenced that the charge densities on the conductors that interact at constant potentials vary with their separation, and one of them can even reverse sign. Moreover, in the NP-electrode interaction, the charge density is not uniform on the surface of the conductors. The redistribution of charge also contributes to extend the region of attraction slightly on the regions where the NP and the electrode have the same charge sign. The regions of attraction and repulsion between the Au electrode and a AgNP (*E*_pzc_ = –0.5 V) are highlighted in Fig. 3C. The attraction of conductors of dissimilar potentials but of the same sign is known both in the presence^27,43,44^ (see ESI† for a detailed explanation) and in the absence^45,46^ of electrolyte solutions. Since the electrolyte solution effectively screens the charge and minimizes the charge redistribution, the NP feels the repulsive force after the potentials cross a certain threshold; the behaviour in vacuum is qualitatively different.^45,46^ This threshold depends on the pzc difference between the NP and the electrode (Fig. 3B). The charge distributions on the electrode and the NP are shown in the ESI.† This redistribution of charge in either the NP or the electrode close to its pzc leads to a situation where part of the surface has positive charge and part of the surface is negatively charged, and this effect gets stronger with increasing the pzc difference of the two materials.

Fig. 3B shows that pzc difference between the NP and the electrode material will have a significant influence on the electrostatic interaction between the NP and the electrode. Hence, the observed differences of the AgNP oxidation when changing the electrode material from Au to GC^16^ could be partly explained by this change in the electrostatic interactions. Fig. 3C shows also that positively charged AgNPs should not be able to approach close enough to the Au or GC electrode to be oxidized upon impact. In reality, the AgNPs are covered with a capping agent like citrate or tannic acid. These capping agents are adsorbed in the inner Helmholtz layer, screen the electrostatic effects of the positively charged core, and in some cases NPs covered with capping agents appear to have a negative charge (as measured with ζ-potential).^7^ Additionally, the double layer models used in this work do not consider specific adsorption of ions in the Stern layer.

All these effects add excess negative charge on the AgNP, resulting in attraction with the positively polarized electrode, and oxidation upon impact. However, if the contact with the electrode is enough to make the total charge of the NP positive enough so that is feels a repulsive force, then the NP will move away from the surface, resulting in a loss of reactivity as its capacitance decreases.

## Conclusions

We have shown that the electric double layer of the electrode increases the differential capacitance of a NP close to the electrode. This means that the outer potential of the NP decreases when the NP moves from the vicinity of the electrode into the bulk (for negative polarization, the outer potential increases). The change in the potential depends on the capacitance of the NP, but also on the size: NPs of above 10 nm radius barely feel any effect, while smaller particles undergo larger changes in potential. However, the effect decreases if higher supporting electrolyte concentrations are used. This means that the NPs are more reactive for oxidation closer to the electrode. For example, 1 nm radius particles will have 30 mV lower potential when moving from the vicinity of an electrode polarized at 0.2 V into the bulk (Fig. 1C). This means also that the surface concentration ratio of the electroactive species will change by a factor of 3. More drastic changes are observed when the NP and the electrode have different potentials of zero charge.

The force between an equilibrated NP and the electrode is always repulsive when they have the same pzc. Otherwise, there is a region of attraction when the NP and the electrode are oppositely charged. However, this region of attraction extends slightly also on the potentials where NP and the electrode have same sign of charge (*i.e.* attraction between two negatively charged or two positively charged objects), because the surface charge redistribution can result in formation of positively charged parts in an overall negatively charged object, and *vice versa*.

In this study, we have used a rather complicated model for the electric double layer, and we expect that the trends of the results will be general. Further improvements could be obtained utilizing more sophisticated methods to describe the double layer structure, and by modelling the electron tunnelling more carefully. However, these results highlight that the effect of the electric double layer of the electrode upon the Fermi level of the NP can be significant, especially with small NPs of different material than the electrode.
