# Correction: Variation of the Fermi level and the electrostatic force of a metallic nanoparticle upon colliding with an electrode

**DOI:** 10.1039/c7sc90040f

**Published:** 2017-06-16

**Authors:** Pekka Peljo, José A. Manzanares, Hubert H. Girault

**Affiliations:** a Laboratoire d’Electrochimie Physique et Analytique (LEPA) , École Polytechnique Fédérale de Lausanne (EPFL) , Rue de l’Industrie 17 , CH-1951 Sion , Switzerland . Email: pekka.peljo@epfl.ch; b Department of Thermodynamics , Faculty of Physics , University of Valencia , c/Dr Moliner, 50 , E-46100 Burjasot , Spain

## Abstract

Correction for ‘Variation of the Fermi level and the electrostatic force of a metallic nanoparticle upon colliding with an electrode’ by Pekka Peljo *et al.*, *Chem. Sci.*, 2017, DOI: ; 10.1039/c7sc00848a.



## 

The authors regret that the incorrect volume and page numbers were provided for ref. 16 and 17 in the original article. The corrected references are listed below as ref. 1 and 2, respectively.

The Royal Society of Chemistry apologises for these errors and any consequent inconvenience to authors and readers.
