# Supplementary material for: Variation of the Fermi level and the electrostatic force of a metallic nanoparticle upon colliding with an electrode
Source: Chem Sci. 2017 May 9;8(7):4795–803. doi: 10.1039/c7sc00848a (PMC5602143; doi:10.1039/c7sc00848a)
Supplement: Supplementary file 1 [file SC-008-C7SC00848A-s001.pdf]

Electronic Supplementary Material (ESI) for Chemical Science.
This journal is © The Royal Society of Chemistry 2017

Electronic Supplementary Information

Variation of the Fermi level and the Electrostatic Force of a Metallic Nanoparticle upon Colliding with an Electrode

Pekka Peljo,[a,]* José A. Manzanares,[b] and Hubert H. Girault[a]

[a] *Laboratoire d'Electrochimie Physique et Analytique (LEPA), Ecole Polytechnique Fédérale de Lausanne (EPFL) Rue de l'Industrie 17, CH-1951 Sion, Switzerland.*
[b] *Department of Thermodynamics, Faculty of Physics, University of Valencia, c/Dr. Moliner, 50, E-46100 Burjasot, Spain.*

**Corresponding author.*
E-mail addresses: pekka.peljo@epfl.ch (Pekka Peljo)

1. Model description and justification of assumptions

The potential distribution around a spherical particle with a radius *r*, separated by a distance *s* from a center of 1 μm radius disk electrode was investigated in an electrolyte solution containing 10 mM KCl, with the two models as described in the main text. In the aqueous electrolyte solution the particle and the electrode were surrounded by an uncharged Stern layer with a radius of 3.3 Å, where the space charge density is nil. The model was solved in 2D axial symmetry ($r = 0$ as the axis of symmetry). Additionally, the electric double layer of a planar electrode was investigated with different models, as shown in Figure S1. The obtained results in 1D agreed well with the analytical solutions, and capacitance calculated for an electrode in a 2D axis symmetry agreed well with the results from the 1D calculations.

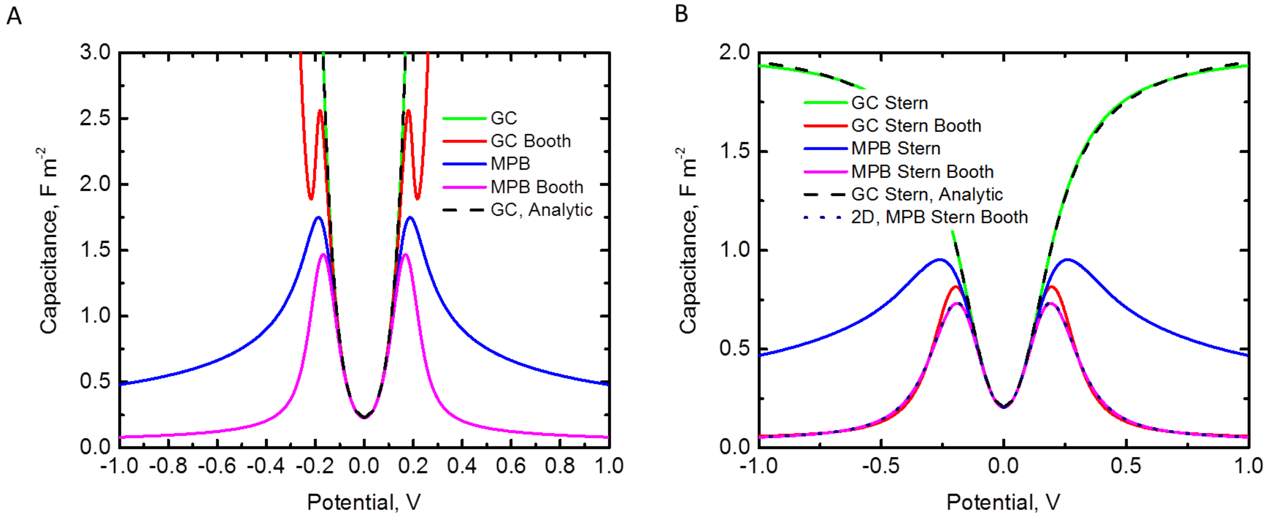

Figure S1. Double layer capacitance of the planar electrode calculated on 1D geometry, A) considering Gouy-Chapman (GC), Gouy-Chapman with Booth (GC Booth) model for relative permittivity of water, modified Poisson-Boltzman model (MPB), also with Booth modification (MPB Booth), and B) Stern modification ($\delta = 0.33$ nm) of all these models. 1:1 electrolyte, 10 mmol L^{-1}.

We have used a modified Poisson-Boltzmann equation that takes into account the finite ion size. This modified Poisson-Boltzmann equation flattens out the electric potential oscillations due to the finite size effects, but the surface charge densities are not expected to be significantly influenced. Moreover, the reliability of continuum models has been studied, e.g., in the context of ion transport through biological ion channels. The comparison of the predictions from the Poisson-Boltzmann equation and from Brownian dynamics has shown that the former are reliable for ion channel radii larger than 1.0 nm.[JA1] Therefore, it is reasonable to accept the validity of the Poisson-Boltzmann equation for the description of the electrical double layer around nanoparticles of radii larger than 1.0 nm.

In this work, the concept of Fermi level equilibration has been applied to the charge transfer between two conductors, the metal NP and the electrode, both immersed in an electrolyte solution. The electrode potential, and hence its Fermi level, is externally fixed by a power supply. The Fermi level is a concept equivalent to the electrochemical potential of the electrons. The electrochemical potential, as the chemical potential, of a species is a thermodynamic quantity that describes the tendency of a system to exchange particles of this species with its surroundings. The energy levels of the particle in the system may also be discrete. *Yet, the chemical potential is not discrete, as it is not necessarily equal to any of the energy*

levels; this is well known in semiconductor electrochemistry. The chemical potential, as the temperature, must be understood as a parameter that determines the probability of occupation of the energy levels of a given species in a system. When the system cannot exchange particles with its surroundings the probability of occupation of the discrete energy levels is only determined by temperature, a continuous variable even though the energy spectrum is discrete. When the system can exchange particles with its surroundings, the probability of occupation of the energy levels is determined by temperature and the chemical potential of the species, both of which are continuous variables. Thus, the equilibration of the chemical potentials of the species in the system and its surroundings is a meaningful concept.

The Fermi level is the electrochemical potential of the electrons in the electrode, $\tilde{\mu}_{e^-}^{E} = -\Phi_E - e\psi'_E$ where is ψ'_E is the electrode potential with respect to vacuum. The Fermi level of the metal nanoparticle in electrolyte solution is $\tilde{\mu}_{e^-}^{NP} = -\Phi_{NP} - e\psi'_{NP}$ where ψ'_{NP} is the nanoparticle potential with respect to vacuum. Although the free energy of the system has not been evaluated, it is implicitly assumed that: (i) it can be considered a function of the nanoparticle charge, $G(Q_{NP})$, (ii) this function can be differentiated with respect to the nanoparticle charge, as if it were a continuous variable, and (iii) the derivative is $-e(\partial G / \partial Q_{NP}) = \tilde{\mu}_{e^-}^{NP} - \tilde{\mu}_{e^-}^{E}$. The condition of Fermi level equilibration is then $\tilde{\mu}_{e^-}^{NP} = \tilde{\mu}_{e^-}^{E}$, or $\psi_{NP} + E_{pzc} = \psi_E$, eqn 20, where ψ_E is the electrode potential with respect to its potential of zero charge (pzc) and E_{pzc} is the pzc of the metal nanoparticle with respect to the electrode pzc. This approach considers the NP as a metallic phase with an outer potential that can vary continuously.

Actually, the NP charge is a discrete variable as it is not possible to exchange a fraction of an electron. This implies that it might not be able to take the value that minimizes $G(Q_{NP})$. In the case we were interested in the electron transfer kinetics, the transfer should be described as a stochastic process according to the orthodox theory.[JA2-JA4] In this theory, the probabilities of electron transfer from the nanoparticle to the electrode, and vice versa, are determined by the free energy changes associated with the transfers, temperature and the tunneling resistance. For a given nanoparticle-electrode separation this theory allows us to evaluate the time average value of the discrete variable Q_{NP}. This time average value is not necessarily one of the discrete values of Q_{NP}.[JA2] At very low temperatures, $k_B T \ll e^2 / 2C_{NP}$, the time average value of Q_{NP} shows the Coulomb staircase. On the contrary, at high temperatures, $k_B T \gg e^2 / 2C_{NP}$, the charge transfer rate is high and the time average value of Q_{NP} is close to the one that makes $G(Q_{NP})$ minimum, $(\partial G / \partial Q_{NP})\big|_{Q_{NP}=Q_{NP,eq}} = 0$. The effects of the discreteness of charge are not seen in this temperature range. The consideration of ψ_{NP} and Q_{NP} as continuous variables is justified by the fact that we do not observe a single NP at a given instant but an average over time.

Alternatively, the consideration of continuous variables can be justified by the fact that we do not observe a single NP but the average behavior of a collection of NPs in solution interacting with the electrode. The NPs are then considered as “molecules” with multiple redox states whose formal redox potentials are equally spaced. The condition of electrochemical equilibrium or Fermi level equilibration for the reaction $NP^z(aq) + e^-(E) \leftrightarrow NP^{z-1}(aq)$, where E stands for metal electrode, is $\tilde{\mu} := \tilde{\mu}_{e^-}^{E} = \tilde{\mu}_{NP^{z-1}}^{aq} - \tilde{\mu}_{NP^z}^{aq}$ or $E = E^{\circ}_{red(z \to z-1)} + (kT/e)\ln(a_{NP^z} / a_{NP^{z-1}})$, for any *z*. The body of work from

R. W. Murray and others[JA5] demonstrating that electron transfer to small nanoparticles is quantized is related to two facts: (1) $E^\circ_{\mathrm{red}(z\to z-1)} \approx E^\circ_{\mathrm{pzc}} + (2z-1)e/2C_{\mathrm{NP}}$ is a function of *z*, and (2) for very small nanoparticles (often, in low relative permittivity solvents) the effective capacitance C_{NP} is so small that the values of the standard redox potentials can be observed individually in a DPV. Since quantized charging seems to be incompatible with the assumption of continuous NP charge, we describe next the conditions under which this assumption is valid.

The NP and the electrode are considered as two systems that reach equilibrium with respect to the exchange of electrons. The negative charge number of the NP is a counter for the number of electrons in the NP. When the charge number is considered a discrete variable, the average value of *z* is given by

$$\langle z\rangle(T,\tilde{\mu}) = \frac{\sum_z z\exp\{-[\varepsilon(z)+z\tilde{\mu}]/kT\}}{\sum_z \exp\{-[\varepsilon(z)+z\tilde{\mu}]/kT\}}. \qquad \text{(JA1)}$$

The ratio of probabilities of observing the charge numbers *z* and *z* – 1 is equal to the ratio of concentrations (or activities) of NPs with these charge numbers

$$\frac{c_z}{c_{z-1}} = \exp\{[\varepsilon(z-1)-\varepsilon(z)-\tilde{\mu}]/kT\}. \qquad \text{(JA2)}$$

The average charge number can also be evaluated in terms of the electrode potential *E*. The comparison of eqn (JA2) with the Nernst equation

$$E = E^\circ_{\mathrm{red}(z\to z-1)} + (kT/e)\ln(c_z/c_{z-1}) \qquad \text{(JA3)}$$

evidences the correspondence

$$\varepsilon(z-1)-\varepsilon(z)-\tilde{\mu} = e[E-E^\circ_{\mathrm{red}(z\to z-1)}]. \qquad \text{(JA4)}$$

Assuming a constant NP capacitance, the standard redox potential can be approximated by

$$E^\circ_{\mathrm{red}(z\to z-1)} = E_{\mathrm{pzc}} + (2z-1)e/2C_{\mathrm{NP}} \qquad \text{(JA5)}$$

where E_{pzc} is the NP potential of zero charge (with respect to that of the electrode), which basically arises from

$$\varepsilon(z) = z^2e^2/2C_{\mathrm{NP}} + z(eE_{\mathrm{pzc}}+k) \qquad \text{(JA6)}$$

and

$$\tilde{\mu} = -eE-k, \qquad \text{(JA7)}$$

where *k* is an arbitrary constant. Substituting eqns (JA6) and (JA7) in (JA1), the average value of *z* can be expressed as in terms of temperature and the electrode potential as

$$\langle z\rangle = \frac{\sum_z z\alpha^z\kappa^{z^2}}{\sum_z \alpha^z\kappa^{z^2}} \qquad \text{(JA8)}$$

where $\alpha := \exp[-e(E_{\mathrm{pzc}}-E)/kT]$ and $\kappa := \exp(-e^2/2kTC_{\mathrm{NP}})$.

The sum in eqn (JA8) cannot be evaluated analytically but there is an alternative procedure to evaluate the (average) charge number as a function of the electrode potential E which is basically equivalent to a mean field approximation. The NP is considered as an isolated system with a charge $\tilde{z}$; the tilde denotes its continuous character. Similarly to eqn (JA6), the NP energy is $\varepsilon(\tilde{z}) = \tilde{z}^2 e^2 / 2C_{\mathrm{NP}} + \tilde{z}(eE_{\mathrm{pzc}} + k)$. The relation between $\tilde{z}$ and E

$$E = E_{\mathrm{pzc}} + \tilde{z}e / C_{\mathrm{NP}} \tag{JA9}$$

is now obtained from eqn (JA7) with the electrochemical potential of the electrons in the NP evaluated from the variation of $\varepsilon(\tilde{z})$ with respect to its number of electrons (i.e., the negative z) as $\tilde{\mu} = -\mathrm{d}\varepsilon / \mathrm{d}\tilde{z} = -\tilde{z}e^2 / C_{\mathrm{NP}} - eE_{\mathrm{pzc}} - k$.

Although only discrete values of the NP charge number are allowed, the average charge number in eqn (JA8) describes the state of the NP solution. In Figure S2, this average value has been represented against $-\ln\alpha$ for different values of $\ln\kappa$ and compared to the continuous charge number in eqn (JA9), here transformed to

$$\tilde{z} = \frac{e(E - E_{\mathrm{pzc}}) / kT}{e^2 / kTC_{\mathrm{NP}}} = -\frac{\ln\alpha}{2\ln\kappa} . \tag{JA10}$$

The sum in eqn (JA8) runs over integer values of z and extends from an arbitrary large negative value to a large positive value; the plot in Figure S2 is restricted to a region when the limits of the sum are irrelevant. When the charging energy $e^2 / 2C_{\mathrm{NP}}$ is larger than ca. 2.5 times the thermal energy kT the average charge number shows the Coulomb staircase. On the contrary, the average charge number shows a linear dependence on $-\ln\alpha = e(E_{\mathrm{pzc}} - E) / kT$ when $e^2 / 2kTC_{\mathrm{NP}}$ is lower than 2.5. The mean field or continuous approximation is then very accurate under the latter conditions, while it also predicts the correct slope of the staircase when $e^2 / 2kTC_{\mathrm{NP}} > 2.5$. The NP capacitances involved in this work are relatively large because we are modeling NPs with diameters between 2 nm and 20 nm, in aqueous solution and without taking into account the protecting monolayer as a low-permittivity dielectric shell. The thermal energy kT at room temperature is then larger that the charging energy, $e^2 / 2C_{\mathrm{NP}}$, and therefore it is reasonable to consider the NP charge number as a continuous variable and eqn (JA10) can be used instead of eqn (JA8), as no Coulomb staircase appears under these conditions.

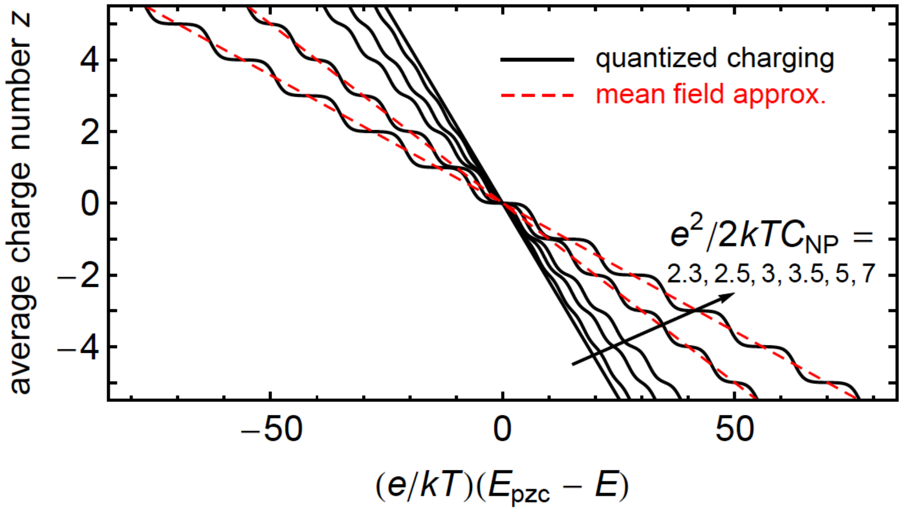

Figure S2. The variation of the average NP charge number with the electrode potential shows the Coulomb staircase when the charging energy $e^2/2C_{NP}$ is a few times larger than the thermal energy kT; the spacing of the ticks in the ordinate scale is one unit. The assumption of the NP charge as a continuous variable is equivalent to a mean field approximation. This approximation is very accurate when $e^2/2kTC_{NP}$ is lower than ca. 2.5. For larger values of this ratio, the mean field approximation (dashed lines, only two are shown for the sake of clarity) does not show the quantized charging but still predicts the correct slope for the variation of charge number with electrode potential.

The restriction to constant NP capacitance has allowed us to clearly establish the conditions under which the average NP charge number does not exhibit quantized charging (i.e. the Coulomb staircase). In most theoretical studies, the NP capacitance is estimated from relatively simple electrostatic models. In this work, the NP capacitance has been calculated from the numerical solution of a modified Poisson-Boltzmann equation that takes into account the finite ion size and the dielectric saturation effect, in addition to the presence of a Stern layer. Obviously, for the purpose of evaluating the differential NP capacitance, the electric charge on the NP must be assumed to be a continuous quantity. Furthermore, when the NP capacitance is not known, the condition of Fermi level equilibration should not be expressed in terms of this capacitance, as in eqn (JA9), but rather in terms of the electric potentials, as in eqn (20), $\psi_E = E_{pzc} + \psi_{NP}$.

The electrochemical equilibrium between a metal electrode with fixed $\tilde{\mu}$ and the solution containing NPs is achieved through charge transfer. This transfer changes the concentrations of NPs in different redox states until their fractions are given by eqn (JA2), the Fermi level equilibration condition. Equation (JA2) essentially says that the transfer of a single electron from the electrode to one NP with charge number z causes a dramatic change: the NP is transformed to a reduced state with charge number $z-1$ and its electrochemical potential changes discretely from $\tilde{\mu}^{aq}_{NP^z}$ to $\tilde{\mu}^{aq}_{NP^{z-1}}$, with

$$\tilde{\mu}^{aq}_{NP^z} - \tilde{\mu}^{aq}_{NP^{z-1}} = \varepsilon(z) - \varepsilon(z-1) + kT \ln \frac{c_z}{c_{z-1}}. \qquad \text{(JA11)}$$

Yet, the electrochemical potential of the electrons in the solution of NPs, $\tilde{\mu} := \tilde{\mu}^{\text{aq}}_{\text{NP}^{z-1}} - \tilde{\mu}^{\text{aq}}_{\text{NP}^{z}}$ for any z, does not undergo any dramatic change due to this electron transfer. The latter is a property of the solution and not as a property of a single NP. Moreover, $\tilde{\mu}$ is not equal to any of the allowed NP energies given by eqn (JA6) with discrete z. The fact that the electrochemical potential $\tilde{\mu}$ of the electrons in the solution of NPs is a continuous variable makes it possible to make it equal to the electrochemical potential of the electrons in the electrode. The latter statement is valid regardless of whether the Coulomb staircase is observable.

2. Schematic description of the Fermi level changes upon NP collision with an electrode and supplementary figures

Schemes S1 and S2 consider the case of electrode and NP made of the same metal $(E_{\text{pzc}} = 0)$, for electrode potential equal ($\psi_{\text{E}} = 0$, Scheme S1) and higher ($\psi_{\text{E}} > 0$, Scheme S2) than the common pzc.

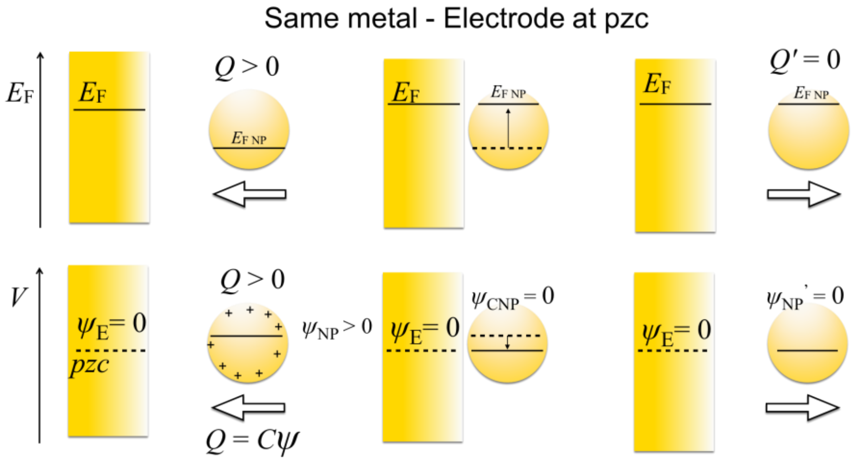

Scheme S1. Electrode and NP made of the same metal. Top panel: Fermi levels. Bottom panel: potential difference between metal and solution. The electrode is at the common pzc. Before collision, the NP is positively charged and, therefore, has a lower Fermi level than the electrode. As the NP capacitance varies with the distance to the electrode, the potential difference between the NP and the solution varies when the NP approaches the electrode. Upon collision the potentials reach the same value, and the NP potential does not change when it moves into the bulk of the solution.

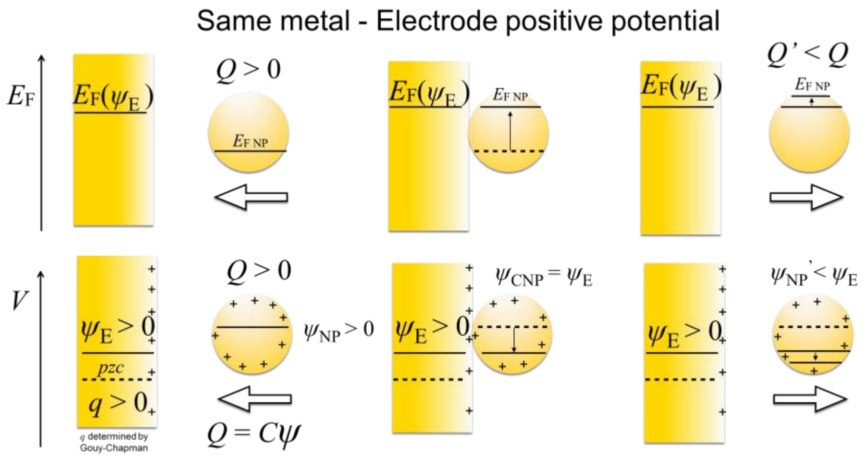

Scheme S2. Similar to Scheme S1, with the electrode at a positive potential. Before collision, the NP is positively charged and has a lower Fermi level than the electrode. As the NP capacitance varies with the distance to the electrode, the potential difference between the NP and the solution varies both when the NP approaches the electrode and when it departs from the electrode after collision

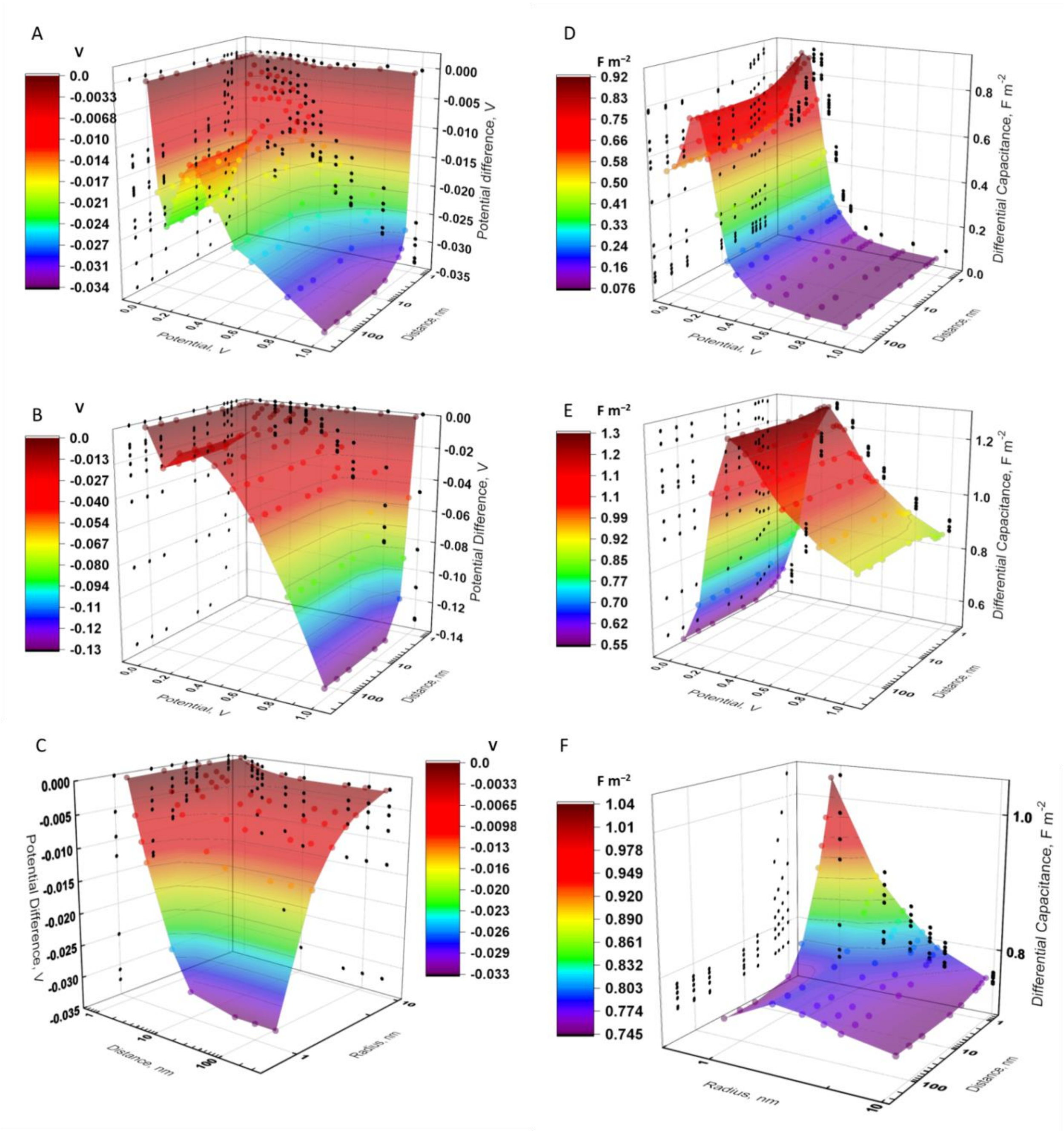

Figure S3. Potential difference between the NP and the electrode after collision as a function of the NP distance and electrode potential for $R_{\mathrm{NP}} = 2\,\mathrm{nm}$, calculated: A) with $\varepsilon_{\mathrm{r}} = 78$ (model I) and B) with the Booth model for the relative permittivity (model II). C) Effect of the NP radius on the differential capacitance at 0.2 V calculated with the Booth model. Differential capacitance at the electrode potential as a function of the NP distance and electrode potential for $R_{\mathrm{NP}} = 2\,\mathrm{nm}$, calculated: D) with $\varepsilon_{\mathrm{r}} = 78$ (model I) and E) with the Booth model for the relative permittivity (model II). F) Effect of the NP radius on the differential capacitance at 0.2 V calculated with the Booth model.

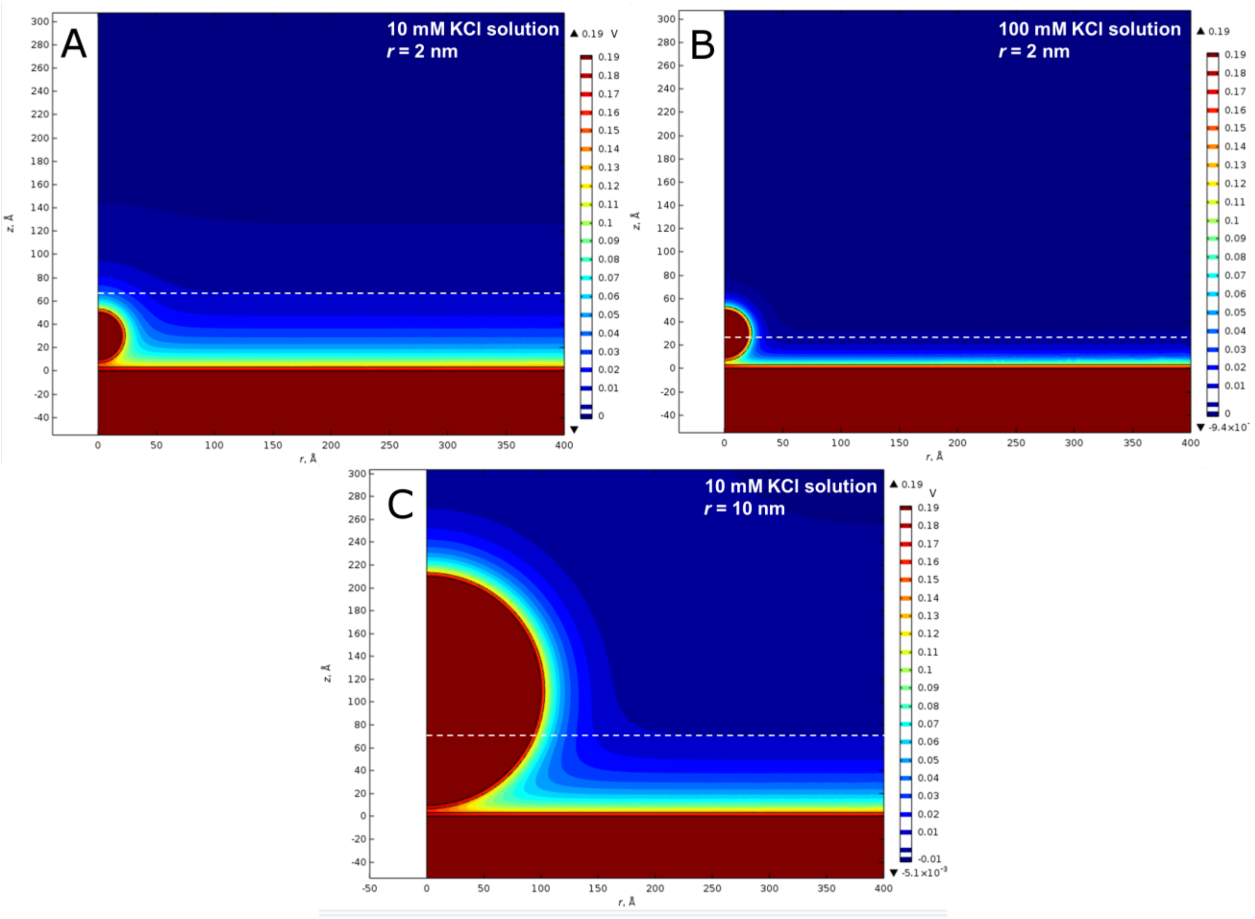

Figure S4. The immersion of the NP in the electric double layer of the electrode at 0.2 V. Small NPs (R = 2 nm) are completely immersed in the double layer in dilute electrolyte solutions (A), but the thickness of the double layer decreases in more concentrated electrolyte solutions (B). The effect of the electric double layer is smaller on larger particles, as they only partially feel the effects of the double layer even in dilute electrolyte solutions (C). s = 1 nm, calculated with Model II. All assuming no specific adsorption of chloride.

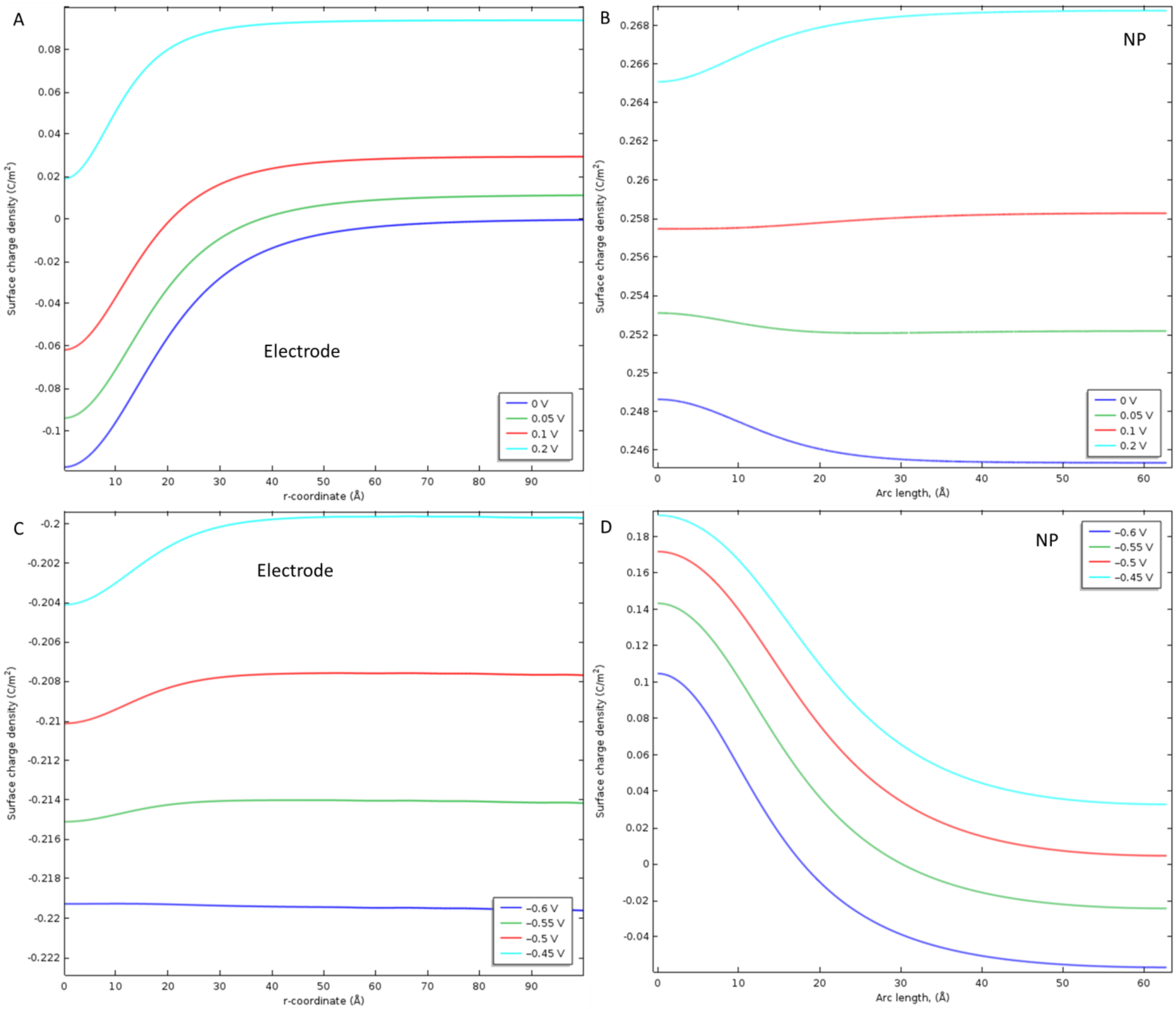

Figure S5. Surface charge densities of the electrode (A, C) and the NP (B, D) for $-E_{pzc} = 0.5V$, for the electrode potentials where the repulsive force changes to attractive (A, B) and again to repulsive (C, D). R_{NP} = 2 nm, s = 1 nm, calculated with model II.

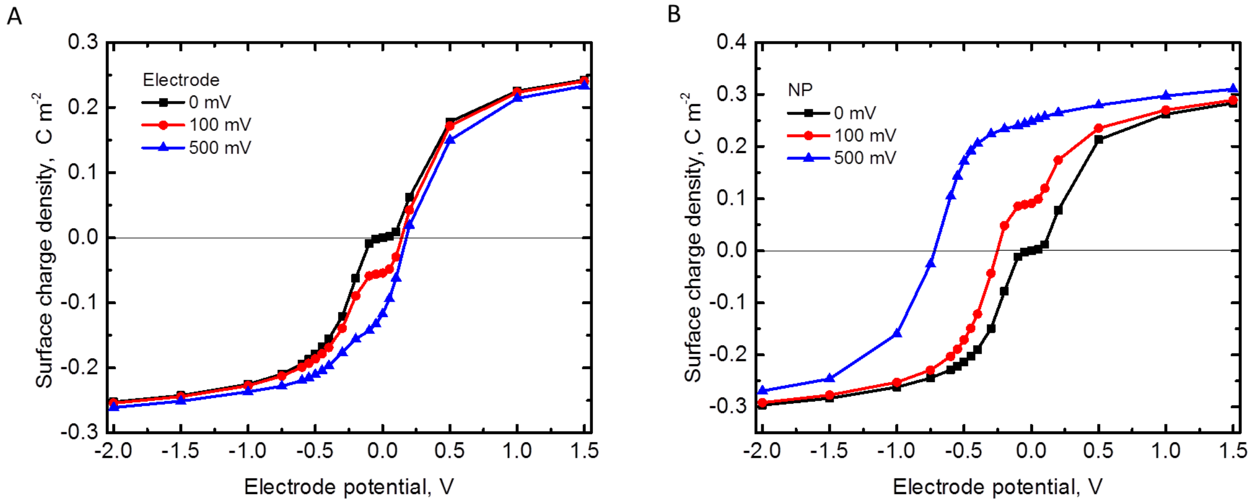

Figure S6. Surface charge density of the electrode at the point closest to the NP (r = 0, z = 0) (A) and the surface charge density at the NP surface at the point closest to the electrode (r = 0, z = 10 Å) (B), as the function of electrode potential, for $-E_{\text{pzc}}$ values of 0, 100 and 500 mV. R_{NP} = 2 nm, s = 1 nm, calculated with model II.

3. Approximate solution of the PBE for a spherical NP with a Stern layer

The main text includes an approximate solution of the PBE for an isolated spherical NP in electrolyte solution with the aim of discussing its differential capacitance. For the sake of simplicity, the approximation discussed is of intermediate complexity. We comment here some details of this approximation as well as another, more accurate approximation. To the best of our knowledge, there are no approximated solutions of the PBE modified to include finite ion size and dielectric saturation effects outside a spherical NP. The results presented in the main text are based on the exact numerical solution of the modified PBE and not in the approximations here discussed for the classical PBE.

The potential distribution inside the Stern layer is

$$\varphi(r) = \varphi(R_{\text{NP}}) + \frac{zF\sigma R_{\text{NP}}}{\varepsilon_0 \varepsilon_{\text{r}} RT}\left(\frac{R_{\text{NP}}}{r} - 1\right) \quad , R_{\text{NP}} \le r \le R_{\text{NP}} + \delta \, . \tag{PBE1}$$

Outside this layer the PBE is approximated by[PBE1]

$$\varphi'' = -\frac{2}{r}\varphi' + \kappa^2 \sinh\varphi \approx \frac{4\kappa}{R_{\text{NP}} + \delta}\sinh\frac{\varphi}{2} + \kappa^2 \sinh\varphi \quad , r \ge R_{\text{NP}} + \delta \, . \tag{PBE2}$$

and integrated with the boundary conditions $\varphi = 0$ and $\varphi' = 0$ when $\kappa r \to \infty$ to yield

$$\frac{1}{\kappa}\varphi' \approx -2\sinh\frac{\varphi}{2}\left[1+\frac{2}{\kappa(R_{\mathrm{NP}}+\delta)}\operatorname{csch}^2\frac{\varphi}{4}\right]^{1/2} \approx -2\sinh\frac{\varphi}{2} - \frac{4}{\kappa(R_{\mathrm{NP}}+\delta)}\tanh\frac{\varphi}{4}\,. \tag{PBE3}$$

The boundary condition $\varphi'(R_{\mathrm{NP}}+\delta) = -zF\sigma/[\varepsilon_0\varepsilon_{\mathrm{r}}RT(1+\delta/R_{\mathrm{NP}})^2]$ then gives

$$\begin{aligned}\sigma &\approx \frac{\varepsilon_0\varepsilon_{\mathrm{r}}\kappa RT(1+\delta/R_{\mathrm{NP}})^2}{zF}\left[2\sinh\frac{\varphi(R_{\mathrm{NP}}+\delta)}{2}+\frac{4}{\kappa(R_{\mathrm{NP}}+\delta)}\tanh\frac{\varphi(R_{\mathrm{NP}}+\delta)}{4}\right]\\ &= \frac{\varepsilon_0\varepsilon_{\mathrm{r}}RT}{zF}\frac{1+\delta/R_{\mathrm{NP}}}{R_{\mathrm{NP}}}\left[2\kappa(R_{\mathrm{NP}}+\delta)\sinh\frac{\varphi(R_{\mathrm{NP}}+\delta)}{2}+4\tanh\frac{\varphi(R_{\mathrm{NP}}+\delta)}{4}\right]\end{aligned} \tag{PBE4}$$

from which the differential capacitance of the NP and the potential drop in the Stern layer can be evaluated as

$$\begin{aligned}C_{\mathrm{NP}}(R_{\mathrm{NP}}+\delta) &\equiv 4\pi R_{\mathrm{NP}}^2\frac{zF}{RT}\frac{\mathrm{d}\sigma}{\mathrm{d}\varphi(R_{\mathrm{NP}}+\delta)}\\ &= 4\pi\varepsilon_0\varepsilon_{\mathrm{r}}(R_{\mathrm{NP}}+\delta)\left[\kappa(R_{\mathrm{NP}}+\delta)\cosh\frac{\varphi(R_{\mathrm{NP}}+\delta)}{2}+\operatorname{sech}^2\frac{\varphi(R_{\mathrm{NP}}+\delta)}{4}\right]\end{aligned} \tag{PBE5}$$

$$\begin{aligned}\varphi(R_{\mathrm{NP}})-\varphi(R_{\mathrm{NP}}+\delta) &= \frac{zF\sigma}{\varepsilon_0\varepsilon_{\mathrm{r}}RT}\frac{R_{\mathrm{NP}}}{R_{\mathrm{NP}}+\delta}\\ &= \frac{2\delta}{R_{\mathrm{NP}}}\left[\kappa(R_{\mathrm{NP}}+\delta)\sinh\frac{\varphi(R_{\mathrm{NP}}+\delta)}{2}+2\tanh\frac{\varphi(R_{\mathrm{NP}}+\delta)}{4}\right]\end{aligned} \tag{PBE6}$$

which are used in the main text.

A more accurate approximate solution of the PBE outside the Stern layer is [PBE1]

$$\varphi(r) = 4\,\mathrm{artanh}\,Bs + 4\,\mathrm{artanh}\frac{Bs}{1+2\kappa(R_{\mathrm{NP}}+\delta)} \quad \text{or}$$

$$\tanh\frac{\varphi(r)}{4} = 2Bs\frac{1+\kappa(R_{\mathrm{NP}}+\delta)}{1+2\kappa(R_{\mathrm{NP}}+\delta)+B^2s^2} \tag{PBE7}$$

where

$$s \equiv \frac{R_{\mathrm{NP}}+\delta}{r}\exp[-\kappa(r-R_{\mathrm{NP}}-\delta)] \tag{PBE8}$$

and B is determined as a function $\varphi(R_{\mathrm{NP}}+\delta)$ of by solving the algebraic equation

$$\tanh\frac{\varphi(R_{\mathrm{NP}}+\delta)}{4} = 2B\frac{1+\kappa(R_{\mathrm{NP}}+\delta)}{1+2\kappa(R_{\mathrm{NP}}+\delta)+B^2}\,. \tag{PBE9}$$

The surface charge density is then obtained as [PBE1]

$$\sigma \approx \frac{\varepsilon_0 \varepsilon_{\mathrm{r}} RT (1+\delta / R_{\mathrm{NP}})^2}{zF} \varphi'(R_{\mathrm{NP}}+\delta) =$$

$$= \frac{2\varepsilon_0 \varepsilon_{\mathrm{r}} RT}{zF} (1+\delta / R_{\mathrm{NP}})^2 \sinh \frac{\varphi(R_{\mathrm{NP}}+\delta)}{2} \tag{PBE10}$$

$$\times \left[1 + \frac{2}{\kappa(R_{\mathrm{NP}}+\delta)} \operatorname{sech}^2 \frac{\varphi(R_{\mathrm{NP}}+\delta)}{4} + \frac{8}{\kappa^2 (R_{\mathrm{NP}}+\delta)^2} \frac{\ln\left(\cosh \frac{\varphi(R_{\mathrm{NP}}+\delta)}{4} \right)}{\sinh^2 \frac{\varphi(R_{\mathrm{NP}}+\delta)}{2}} \right]^{1/2}$$

and the potential drop in the Stern layer is

$$\varphi(R_{\mathrm{NP}}) - \varphi(R_{\mathrm{NP}}+\delta) = \frac{zF\sigma}{\varepsilon_0 \varepsilon_{\mathrm{r}} RT} \frac{R_{\mathrm{NP}}\delta}{R_{\mathrm{NP}}+\delta} . \tag{PBE11}$$

4. Force between charged plates (PP) at different potentials separated by a *z*:*z* electrolyte solution

In a *z*:*z* electrolyte the Poisson-Boltzmann equation (PBE), eqn (4), can be multiplied by $2\nabla\varphi$ and integrated to give

$$(\nabla\varphi)^2 = 2\kappa^2 (\cosh\varphi - B) \tag{PP1}$$

where B is an integration constant. To describe the interaction between two parallel plates, we note that $\Pi(z) - \varepsilon_0 \varepsilon_{\mathrm{r}} E_z^2(z)/2$ is independent of the position between the plates, where $\Pi(z) = 2RTc^{\mathrm{b}}[\cosh\varphi(z) - 1]$ is the local osmotic pressure measured with respect to its bulk value. The local electric field, eqn (PP2), satisfies

$$E_z^2(z) = \left(\frac{RT}{zF} \right)^2 2\kappa^2 [\cosh\varphi(z) - B] = \frac{4RTc^{\mathrm{b}}}{\varepsilon_0 \varepsilon_{\mathrm{r}}} [\cosh\varphi(z) - B] \tag{PP2}$$

where we have used $\varepsilon_0 \varepsilon_{\mathrm{r}} \kappa^2 (RT/zF)^2 = 2RTc^{\mathrm{b}}$. As required by the mechanical equilibrium, the total stress

$$\Pi(z) - \frac{1}{2} \varepsilon_0 \varepsilon_{\mathrm{r}} E_z^2(z) = 2RTc^{\mathrm{b}}(B-1) \tag{PP3}$$

is indeed independent of position. Therefore, the force on a plate can be determined by evaluating the integration constant *B* in eqn (PP1) from the values of the potential at the plates and their separation.

The integration of eqn (PP1) in planar geometry under arbitrary boundary conditions can be done analytically but involves elliptic integrals or Jacobi elliptic functions.[PP1,PP2] To avoid these complications, it is customary to discuss the interaction between plates using the classical HHF method for the small potentials.[PP3] Consider that the plate located at $z = 0$ has potential φ_{NP} and the plate at $z = s$ has potential φ_{E}. For the sake of simplicity, these dimensionless potentials are both small so that the PBE can be linearized to $\mathrm{d}^2\varphi / \mathrm{d}z^2 = \kappa^2\varphi$. The solution of this equation is

$$\varphi(z) = \varphi_{\mathrm{NP}} \cosh(\kappa z) + \left[\frac{\varphi_{\mathrm{E}}}{\sinh(\kappa s)} - \frac{\varphi_{\mathrm{NP}}}{\tanh(\kappa s)} \right] \sinh(\kappa z) \,. \tag{PP4}$$

Then, the electric field is

$$\frac{1}{\kappa}\frac{\mathrm{d}\varphi}{\mathrm{d}z} = \frac{\varphi_{\mathrm{E}} \cosh(\kappa z) - \varphi_{\mathrm{NP}} \cosh[\kappa(z-s)]}{\sinh(\kappa s)} \tag{PP5}$$

and the constant B is given by

$$2(B-1) = 2\cosh\varphi - 2 - \left(\frac{1}{\kappa}\frac{\mathrm{d}\varphi}{\mathrm{d}z} \right)^2 \approx \varphi^2 - \left(\frac{1}{\kappa}\frac{\mathrm{d}\varphi}{\mathrm{d}z} \right)^2 = \left(\frac{\varphi_{\mathrm{NP}} + \varphi_{\mathrm{E}}}{2\cosh(\kappa s/2)} \right)^2 - \left(\frac{\varphi_{\mathrm{NP}} - \varphi_{\mathrm{E}}}{2\sinh(\kappa s/2)} \right)^2 . \tag{PP6}$$

Thus, we conclude that the total force

$$F(s) = 2RTc^{\mathrm{b}}(B-1)A, \tag{PP7}$$

where A is the plate area, is positive (i. e., repulsive) when $\varphi_{\mathrm{NP}} = \varphi_{\mathrm{E}}$, but it can be attractive when the separation s between the plates is small and their potentials are different, even if they are of the same sign.[PP4,PP5] Consider, without loss of generality, that $\varphi_{\mathrm{NP}} > \varphi_{\mathrm{E}} > 0$. The force reverses from repulsive to attractive when $\tanh(\kappa s/2) = (\varphi_{\mathrm{NP}} - \varphi_{\mathrm{E}})/(\varphi_{\mathrm{NP}} + \varphi_{\mathrm{E}})$, that is, when $\varphi_{\mathrm{E}} = \varphi_{\mathrm{NP}}\,\mathrm{e}^{-\kappa s}$; this is consistent with the arguments made above, as the condition $\varphi_{\mathrm{E}} = 4\mathrm{artanh}[\tanh(\varphi_{\mathrm{NP}}/4)\exp(-\kappa s)]$ for vanishing force mentioned at the beginning of this section corresponds to $\varphi_{\mathrm{E}} \approx \varphi_{\mathrm{NP}}\,\mathrm{e}^{-\kappa s}$ for small potentials. The force between the plates is attractive if $\varphi_{\mathrm{E}} < \varphi_{\mathrm{NP}}\,\mathrm{e}^{-\kappa s}$ and repulsive if $\varphi_{\mathrm{NP}} > \varphi_{\mathrm{E}} > \varphi_{\mathrm{NP}}\,\mathrm{e}^{-\kappa s}$.

The attractive interaction between plates with dissimilar potentials of the same sign corresponds to an attractive interaction between charges densities of opposite sign.[PP6,PP7] The plates have opposite charge densities when $\varphi_{\mathrm{NP}} > \varphi_{\mathrm{E}} \cosh(\kappa s)$, and hence the condition $\varphi_{\mathrm{NP}} > \varphi_{\mathrm{E}}\,\mathrm{e}^{\kappa s}$ for observing an attractive force implies that the plates bear charge densities of opposite sign as $\mathrm{e}^{\kappa s} = \cosh(\kappa s) + \sinh(\kappa s) > \cosh(\kappa s)$. Indeed, the charge density on the plate at $z = 0$ is

$$\sigma_{\mathrm{NP}} = -\varepsilon_0\varepsilon_{\mathrm{r}}\kappa \frac{RT}{zF}\frac{\mathrm{d}\varphi}{\mathrm{dz}}\bigg|_{z=0} = \varepsilon_0\varepsilon_{\mathrm{r}}\kappa \frac{RT}{zF}\frac{\varphi_{\mathrm{NP}}\cosh(\kappa s) - \varphi_{\mathrm{E}}}{\sinh(\kappa s)}$$
$$= \varepsilon_0\varepsilon_{\mathrm{r}}\kappa \frac{RT}{2zF}\left[(\varphi_{\mathrm{NP}} + \varphi_{\mathrm{E}})\tanh(\kappa s/2) + \frac{\varphi_{\mathrm{NP}} - \varphi_{\mathrm{E}}}{\tanh(\kappa s/2)}\right] > 0 \qquad \text{(PP8)}$$

and the charge density on the plate at z = s is

$$\sigma_{\mathrm{E}} = \varepsilon_0\varepsilon_{\mathrm{r}}\kappa \frac{RT}{zF}\frac{\mathrm{d}\varphi}{\mathrm{dz}}\bigg|_{z=s} = \varepsilon_0\varepsilon_{\mathrm{r}}\kappa \frac{RT}{zF}\frac{\varphi_{\mathrm{E}}\cosh(\kappa s) - \varphi_{\mathrm{NP}}}{\sinh(\kappa s)}$$
$$= \varepsilon_0\varepsilon_{\mathrm{r}}\kappa \frac{RT}{2zF}\left[(\varphi_{\mathrm{NP}} + \varphi_{\mathrm{E}})\tanh(\kappa s/2) - \frac{\varphi_{\mathrm{NP}} - \varphi_{\mathrm{E}}}{\tanh(\kappa s/2)}\right]. \qquad \text{(PP9)}$$

The latter vanishes when $\varphi_{\mathrm{NP}} = \varphi_{\mathrm{E}}\cosh(\kappa s)$. Then, the plates have charge densities of opposite sign when $\varphi_{\mathrm{NP}} > \varphi_{\mathrm{E}}\cosh(\kappa s)$. For fixed φ_{E} and φ_{NP}, the repulsive force is maximum at a separation such that $\varphi_{\mathrm{NP}} = \varphi_{\mathrm{E}}\cosh(\kappa s)$, which corresponds to vanishing charge density on the plate at $z = s$.

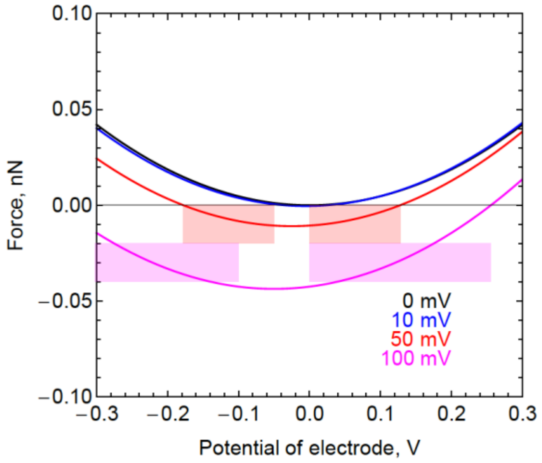

Figure S7. Force $2RTc^{\mathrm{b}}(B-1)A$ between two parallel plates of area $A = \pi R_{\mathrm{NP}}^2$ separated by 1 nm thick layer of a 10 mmol/L 1:1 electrolyte solution. The potential of one plate is indicated in the abscissa axis. Four values (0, 10, 50 and 100 mV) have been considered for the potential difference $(RT/zF)(\varphi_{\mathrm{NP}} - \varphi_{\mathrm{E}})$ between the plates. The shaded regions correspond to ranges of attraction between two plates at potentials of the same sign.

Figure S7 evaluates the force $2RTc^{\mathrm{b}}(B-1)A$ for a typical area A of a 2 nm NP and shows a remarkable agreement with the results shown in Figure 3A-C, which are then partly explained. Moreover, eqn (PP6) explains that repulsion dominates when φ_{NP} and φ_{E} are increased in magnitude while keeping constant $\varphi_{\mathrm{NP}} - \varphi_{\mathrm{E}}$, as observed in Figure 3A-C. Finally, although these results have been derived from the linearized PBE, these conclusions are expected to hold qualitatively for large potentials.

The relation between the surface charge densities and the surface potentials can be presented in matrix form as

$$\begin{pmatrix} \sigma_{\text{NP}} \\ \sigma_{\text{E}} \end{pmatrix} = \varepsilon_0 \varepsilon_{\text{r}} \kappa \begin{pmatrix} \coth(\kappa s) & -\text{csch}(\kappa s) \\ -\text{csch}(\kappa s) & \coth(\kappa s) \end{pmatrix} \begin{pmatrix} \psi_{\text{NP}} \\ \psi_{\text{E}} \end{pmatrix}. \tag{PP10}$$

At large separations, $\kappa s >> 1$, there is no mutual influence and the areal capacitance of the isolated plates is $C_{\text{GC}\infty} = \varepsilon_0 \varepsilon_{\text{r}} \kappa$. In general, the areal capacitance of one plate, $C_{\text{NP}}(s) = \left(\partial \sigma_{\text{NP}} / \partial \psi_{\text{NP}} \right)_{s,\psi_{\text{E}}}$ $= \varepsilon_0 \varepsilon_{\text{r}} \kappa \coth(\kappa s)$, is a decreasing function of *s*.

When the plates interact at constant potentials, the (surface density of) potential energy is

$$\begin{aligned} \tilde{W}(s) &= -\frac{1}{2}\left(\sigma_{\text{NP}} \psi_{\text{NP}} + \sigma_{\text{E}} \psi_{\text{E}} \right) = \frac{\varepsilon_0 \varepsilon_{\text{r}} \kappa}{2} \left[2\psi_{\text{NP}} \psi_{\text{E}} \,\text{csch}(\kappa s) - \left(\psi_{\text{NP}}^2 + \psi_{\text{E}}^2 \right) \coth(\kappa s) \right] \\ &= -\frac{\varepsilon_0 \varepsilon_{\text{r}} \kappa}{4} \left[(\psi_{\text{NP}} + \psi_{\text{E}})^2 \tanh(\kappa s/2) + (\psi_{\text{NP}} - \psi_{\text{E}})^2 \coth(\kappa s/2) \right] \end{aligned} \tag{PP11}$$

and its derivative gives the force $F(s)$ between the plates

$$\begin{aligned} \frac{F(s)}{A} &= -\frac{\mathrm{d}\tilde{W}}{\mathrm{d}s} = \frac{\varepsilon_0 \varepsilon_{\text{r}} \kappa^2}{2} \left[\left(\frac{\psi_{\text{NP}} + \psi_{\text{E}}}{2\cosh(\kappa s/2)} \right)^2 - \left(\frac{\psi_{\text{NP}} - \psi_{\text{E}}}{2\sinh(\kappa s/2)} \right)^2 \right] \\ &= RTc^{\text{b}} \left[\left(\frac{\varphi_{\text{NP}} + \varphi_{\text{E}}}{2\cosh(\kappa s/2)} \right)^2 - \left(\frac{\varphi_{\text{NP}} - \varphi_{\text{E}}}{2\sinh(\kappa s/2)} \right)^2 \right]. \end{aligned} \tag{PP12}$$

By subtracting the energy of the isolated plates, the interaction energy is[PP3,PP8]

$$\begin{aligned} \tilde{W}_{\text{int}} &= -\frac{1}{2}\left(\sigma_{\text{NP}} \psi_{\text{NP}} + \sigma_{\text{E}} \psi_{\text{E}} \right) + \frac{1}{2}\left(\sigma_{\text{NP}\infty} \psi_{\text{NP}} + \sigma_{\text{E}\infty} \psi_{\text{E}} \right) \\ &= \frac{\varepsilon_0 \varepsilon_{\text{r}} \kappa}{2} \left\{ \left(\psi_{\text{NP}}^2 + \psi_{\text{E}}^2 \right) [1 - \coth(\kappa s)] + 2\psi_{\text{NP}} \psi_{\text{E}} \,\text{csch}(\kappa s) \right\} \end{aligned} \tag{PP13}$$

where $\sigma_{\text{E}\infty} = C_{\text{GC}\infty} \psi_{\text{E}}$ and $\sigma_{\text{NP}\infty} = C_{\text{GC}\infty} \psi_{\text{NP}}$ are surface charge densities corresponding to infinite separation.

The linear PBE should not be used to describe the interaction at constant charge for small separations, as the potentials may take then so large values that the PBE cannot be linearized.[PP9]

5. Force between a spherical NP and a planar electrode (SP) at constant potentials in an electrolyte solution

Consider a spherical NP and a planar electrode at a separation s in an electrolyte solution with Debye parameter κ. For large particles ($\kappa R_{\mathrm{NP}} >> 1$) and low potentials ($\varphi << 1$), the interaction potential energy between the NP and the electrode when they are hold at constant potentials is[PP3,SP1]

$$W_{\mathrm{int}}^{\psi}(s) = \frac{C_{\infty}}{4}\left[(\psi_{\mathrm{NP}} + \psi_{\mathrm{E}})^2 \ln\left(1 + \mathrm{e}^{-\kappa s}\right) + (\psi_{\mathrm{NP}} - \psi_{\mathrm{E}})^2 \ln\left(1 - \mathrm{e}^{-\kappa s}\right)\right] \tag{SP1}$$

where $C_{\infty} = 4\pi\varepsilon_0\varepsilon_{\mathrm{r}}R_{\mathrm{NP}}$ is the capacitance of an isolated NP in the absence of electrolyte. Observe that W_{int}^{ψ} is the sum of a positive term proportional to the square of the average potential which describes the potential energy of the NP-electrode system when it is charged as a whole and a negative term proportional to the square of the potential difference that describes the potential energy of a capacitor that uses the NP and the electrode as its “plates”.[SP2]

When the NP and the electrode interact at constant potentials, their potential energy is

$$\begin{aligned} W^{\psi}(s) &= -\frac{1}{2}\left(Q_{\mathrm{NP}}\psi_{\mathrm{NP}} + Q_{\mathrm{E}}\psi_{\mathrm{E}}\right) = W_{\mathrm{int}}^{\psi}(s) - \frac{1}{2}\left(C_{\infty}\psi_{\mathrm{NP}}^2 + C_{\mathrm{GC}\infty}A\psi_{\mathrm{E}}^2\right) \\ &= \frac{C_{\infty}}{4}\left[(\psi_{\mathrm{NP}}^2 + \psi_{\mathrm{E}}^2)\ln\left(1 - \mathrm{e}^{-2\kappa s}\right) + 2\psi_{\mathrm{NP}}\psi_{\mathrm{E}} \ln\frac{1 + \mathrm{e}^{-\kappa s}}{1 - \mathrm{e}^{-\kappa s}}\right] - \frac{1}{2}\left(C_{\infty}\psi_{\mathrm{NP}}^2 + C_{\mathrm{GC}\infty}A\psi_{\mathrm{E}}^2\right) \\ &= -\frac{1}{2}\left(C_{\mathrm{NP,NP}}\psi_{\mathrm{NP}}^2 + C_{\mathrm{E,E}}\psi_{\mathrm{E}}^2 + 2C_{\mathrm{NP,E}}\psi_{\mathrm{NP}}\psi_{\mathrm{E}}\right) \end{aligned} \tag{SP2}$$

where in the last step we have introduced the capacitance matrix coefficients and $C_{\mathrm{GC}\infty} = \varepsilon_0\varepsilon_{\mathrm{r}}\kappa$. The charge on the NP is

$$Q_{\mathrm{NP}}(s) = C_{\mathrm{NP,NP}}\psi_{\mathrm{NP}} + C_{\mathrm{NP,E}}\psi_{\mathrm{E}} = C_{\infty}\left[1 - \frac{1}{2}\ln\left(1 - \mathrm{e}^{-2\kappa s}\right)\right]\psi_{\mathrm{NP}} - \frac{C_{\infty}}{2}\ln\frac{1 + \mathrm{e}^{-\kappa s}}{1 - \mathrm{e}^{-\kappa s}}\psi_{\mathrm{E}}. \tag{SP3}$$

The force between the NP and the electrode is

$$F(s) = -\frac{\mathrm{d}W_{\mathrm{int}}^{\psi}}{\mathrm{d}s} = \frac{C_{\infty}\kappa}{4}\left[\frac{(\psi_{\mathrm{NP}} + \psi_{\mathrm{E}})^2}{\mathrm{e}^{\kappa s} + 1} - \frac{(\psi_{\mathrm{NP}} - \psi_{\mathrm{E}})^2}{\mathrm{e}^{\kappa s} - 1}\right] \tag{SP4}$$

where the first term describes a repulsive contribution and the second one an attractive contribution that dominates at short separations, i.e. if $\mathrm{e}^{\kappa s} < (\psi_{\mathrm{NP}}^2 + \psi_{\mathrm{E}}^2)/2\psi_{\mathrm{NP}}\psi_{\mathrm{E}}$. The similarity between eqns (PP6) and (SP4) is not casual, as the force between a sphere and a plane is closely related to the force between two planar surfaces.[SP3,PP8] In the limit of large separations ($\kappa s >> 1$) this force reduces to

$$F_{\infty}(s) \approx C_{\infty}\kappa\psi_{\mathrm{NP}}\psi_{\mathrm{E}}\mathrm{e}^{-\kappa s} \tag{SP5}$$

and in the absence of electrolyte solution the force is attractive and reduces to

$$F_{\kappa\to 0}(s) = -\frac{C_\infty}{4s}(\psi_{\rm NP} - \psi_{\rm E})^2 \qquad \text{(SP6)}$$

In the case of NPs with smaller radii, i.e. for arbitrary $\kappa R_{\rm NP}$, and low potentials ($\varphi << 1$), the interaction potential energy between the NP and the electrode when they are hold at constant potentials is[PP8]

$$W^*_{\rm int}(s) = W^\psi_{\rm int}(s) + W^\psi_{\rm int}(s + 2R_{\rm NP}) + \beta(s) - \beta(s + 2R_{\rm NP}) \qquad \text{(SP7)}$$

where $W^\psi_{\rm int}(x)$ is given by eqn (SP1),

$$\beta(x) = \frac{C_\infty}{4\kappa R_{\rm NP}}\left[(\psi_{\rm NP} + \psi_{\rm E})^2 {\rm Li}_2(-{\rm e}^{-\kappa x}) + (\psi_{\rm NP} - \psi_{\rm E})^2 {\rm Li}_2({\rm e}^{-\kappa x})\right] \qquad \text{(SP8)}$$

is a correction function defined from the condition $W^\psi_{\rm int}(x) = R_{\rm NP}{\rm d}\beta / {\rm d}x$, and ${\rm Li}_2(z) = \sum_{k=1}^\infty z^k / k^2$ is a polylogarithm function whose derivative is ${\rm dLi}_2(z)/{\rm d}z = -\ln(1-z)/z$. The force between the NP and the electrode is then

$$F^*(s) = -\frac{{\rm d}W^*_{\rm int}}{{\rm d}s} = \frac{C_\infty \kappa}{4}(\psi_{\rm NP} + \psi_{\rm E})^2\left(\frac{1}{{\rm e}^{\kappa s}+1} + \frac{1}{{\rm e}^{\kappa(s+2R_{\rm NP})}+1} + \frac{1}{\kappa R_{\rm NP}}\ln\frac{{\rm e}^{-\kappa(s+2R_{\rm NP})}+1}{{\rm e}^{-\kappa s}+1}\right)$$
$$-\frac{C_\infty \kappa}{4}(\psi_{\rm NP} - \psi_{\rm E})^2\left(\frac{1}{{\rm e}^{\kappa s}-1} + \frac{1}{{\rm e}^{\kappa(s+2R_{\rm NP})}-1} + \frac{1}{\kappa R_{\rm NP}}\ln\frac{{\rm e}^{-\kappa s}-1}{{\rm e}^{-\kappa(s+2R_{\rm NP})}-1}\right). \qquad \text{(SP9)}$$

This force is repulsive if $\psi_{\rm NP} = \psi_{\rm E}$, but it can be attractive at short separations if $\psi_{\rm NP} \neq \psi_{\rm E}$. In the limit of large separations ($\kappa s >> 1$) the force reduces to

$$F^*_\infty(s) = C_\infty \kappa \psi_{\rm NP}\psi_{\rm E}{\rm e}^{-\kappa s}\left(1 + {\rm e}^{-2\kappa R_{\rm NP}} + \frac{1-{\rm e}^{-2\kappa R_{\rm NP}}}{\kappa R_{\rm NP}}\right) \qquad \text{(SP10)}$$

and in the absence of electrolyte solution the force is attractive and reduces to

$$F^*_{\kappa\to 0}(s) = -\frac{C_\infty}{4}(\psi_{\rm NP} - \psi_{\rm E})^2\left[\frac{1}{s} + \frac{1}{s+2R_{\rm NP}} - \frac{\ln(1+2R_{\rm NP}/s)}{R_{\rm NP}}\right]. \qquad \text{(SP11)}$$

The sphere-plate interaction in electrolyte solutions has been discussed in a number of papers, most of them considering the small potential approximation;[SP4-SP10] the case of metal sphere and metal plate must sometimes be obtained by taking the limit of relative permittivity (of the sphere and the plate) tending to infinity. Ohshima has discussed an exact solution for the plate-sphere interaction that is based on a generalization of the method of image charges.[SP11,SP12] Unfortunately, the expression obtained in terms of series expansions is so complicated that has very limited practical value. The important remark

from Ohshima work, however, is that the image charges always contribute with an attractive term to the interaction force between the sphere and the plate.[SP12]

6. Solution of the Laplace equation (LE) in the sphere-plate capacitor

In the absence of electrolyte the potential distribution satisfies the Laplace equation $\nabla^2\psi = 0$, which can be solved analytically in the space between the conducting surfaces of a sphere-plate capacitor. The electric field distribution is determined by the boundary conditions and the potential is defined up to an arbitrary constant. That is, only the potential difference between the sphere (or nanoparticle NP) and the plate (or electrode E) is relevant. For this reason, the description of the electrostatics of the sphere-plate interaction often considers that the plate is grounded. Should the plate potential be different, its value should be added to the potential distribution described below; with the correct potential difference between sphere and plate. In electrostatics it is well known that for a conducting sphere approaching a conducting plane, the mutual capacitance coefficient is[LE1,LE2] $C_{\text{NP,E}} = -C_{\text{NP,NP}}$ and, therefore, $Q_{\text{NP}} = C_{\text{NP,NP}}\psi_{\text{NP}} + C_{\text{NP,E}}\psi_{\text{E}} = C_{\text{NP,NP}}(\psi_{\text{NP}} - \psi_{\text{E}})$, which is reduced to $Q_{\text{NP}} = C_{\text{NP}}\psi_{\text{NP}}$ if $\psi_{\text{E}} = 0$.

The centre of the NP of radius R_{NP} is located on the Cartesian z axis at $z_{\text{centre}} = s + R_{\text{NP}}$, where s is the separation between NP and electrode. Hereinafter, a tilde ~ denotes division by R_{NP}; e. g., $\tilde{s} \equiv s / R_{\text{NP}}$. Using bispherical coordinates (η,θ,ϕ),[LE3, LE4] the surface $\eta = \eta_0(\tilde{s})$ with

$$\eta_0(\tilde{s}) = \text{arcosh}(1+\tilde{s}) = \ln\left[1+\tilde{s}+(\tilde{s}^2+2\tilde{s})^{1/2}\right] \tag{LE1}$$

is spherical and corresponds to the NP surface; obviously, $z_{\text{centre}} = R_{\text{NP}}\cosh\eta_0$. The surface $\eta = 0$ is planar and corresponds to the plate ($z = 0$ in Cartesian coordinates); the origin of coordinates is the plate position closest to the NP. The space between the sphere and the plate is ($0 \le \eta \le \eta_0(\tilde{s})$, $0 \le \theta \le \pi$, $0 \le \phi \le 2\pi$). The interior of the NP is $\eta > \eta_0(\tilde{s})$.

The solution of the Laplace equation is

$$\psi(\tilde{s},\eta,\theta) = \psi_{\text{NP}} 2^{3/2}(\cosh\eta - \cos\theta)^{1/2} \sum_{m=0}^{\infty} \frac{\sinh[(m+1/2)\eta]}{\exp[(2m+1)\eta_0(\tilde{s})]-1} P_m(\cos\theta) \tag{LE2}$$

The electric field is

$$\mathbf{E} = -\nabla\psi = -\frac{\cosh\eta - \cos\theta}{a(\tilde{s})}\left(\frac{\partial\varphi}{\partial\eta}\mathbf{e}_\eta + \frac{\partial\varphi}{\partial\theta}\mathbf{e}_\theta\right) = -\frac{\cosh\eta - \cos\theta}{a(\tilde{s})}\left(\frac{\partial\varphi}{\partial\eta}\mathbf{e}_\eta - \sin\theta\frac{\partial\varphi}{\partial\cos\theta}\mathbf{e}_\theta\right) \tag{LE3}$$

where

$$\tilde{a}(\tilde{s}) \equiv a / R_{\text{NP}} = \sinh\eta_0 = (\tilde{s}^2 + 2\tilde{s})^{1/2} \tag{LE4}$$

is a scale parameter of the bispherical coordinates. The electric field only has η component at the surfaces of the sphere and the plate, as the field is normal to these conducting surfaces and they are both constant η surfaces, and hence normal to the bispherical unit vector $\mathbf{e}_\eta$; at the plate $\mathbf{e}_\eta$ points in the positive *z* direction and at the NP it points towards the inside of the NP. The η component of the field is

$$E_\eta = -\frac{\psi_{\mathrm{NP}}}{R_{\mathrm{NP}}}\frac{2^{3/2}(\cosh\eta-\cos\theta)^{1/2}}{\sinh\eta_0}\sum_{m=0}^{\infty}\frac{P_m(\cos\theta)}{\exp[(2m+1)\eta_0]-1} \times\left\{\frac{1}{2}\sinh\eta\sinh[(m+1/2)\eta]+(m+1/2)(\cosh\eta-\cos\theta)\cosh[(m+1/2)\eta]\right\}. \tag{LE5}$$

At the conducting plate (*z* = 0 and $\eta = 0$) the field is

$$\begin{aligned} E_{\mathrm{plate},z}(\tilde{s},\theta) &= -\frac{\psi_{\mathrm{NP}}}{R_{\mathrm{NP}}}\frac{2^{3/2}(1-\cos\theta)^{3/2}}{\sinh\eta_0}\sum_{m=0}^{\infty}\frac{(m+1/2)P_m(\cos\theta)}{\exp[(2m+1)\eta_0]-1} \\ &= -\frac{\psi_{\mathrm{NP}}}{R_{\mathrm{NP}}}\frac{8\sin^3(\theta/2)}{(\tilde{s}^2+2\tilde{s})^{1/2}}\sum_{m=0}^{\infty}\frac{(m+1/2)P_m(\cos\theta)}{[1+\tilde{s}+(\tilde{s}^2+2\tilde{s})^{1/2}]^{2m+1}-1} \end{aligned} \tag{LE6}$$

which can be represented (parametrically in θ) against the distance $\rho(\theta) = a/\tan(\theta/2)$ along the plate to the Cartesian origin for any value of the dimensionless separation $\tilde{s} \equiv s/R_{\mathrm{NP}}$. Since the charge density on the plate is $\sigma(\tilde{s},\theta) = \varepsilon_0\varepsilon_{\mathrm{r}}E_{\mathrm{plate},z}$, the total charge on the plate is

$$\begin{aligned} Q_{\mathrm{plate}} &= \varepsilon_0\varepsilon_{\mathrm{r}}\int_0^\infty E_{\mathrm{plate},z}2\pi\rho d\rho = \pi\varepsilon_0\varepsilon_{\mathrm{r}}a^2\int_0^{\pi}E_{\mathrm{plate},z}\frac{\cos(\theta/2)}{\sin^3(\theta/2)}\mathrm{d}\theta \\ &= -\psi_{\mathrm{NP}}\pi\varepsilon_0\varepsilon_{\mathrm{r}}R_{\mathrm{NP}}\sinh\eta_0\sum_{m=0}^{\infty}\frac{8(m+1/2)}{\exp[(2m+1)\eta_0]-1}\int_0^{\pi}P_m(\cos\theta)\cos(\theta/2)\mathrm{d}\theta \\ &= -\psi_{\mathrm{NP}}2C_\infty\sinh\eta_0\sum_{m=0}^{\infty}\frac{1}{\exp[(2m+1)\eta_0]-1} = -C_{\mathrm{NP}}(\tilde{s})\psi_{\mathrm{NP}} \end{aligned} \tag{LE7}$$

where $C_\infty = 4\pi\varepsilon_0\varepsilon_{\mathrm{r}}R_{\mathrm{NP}}$ is the capacitance of the isolated NP. The capacitance of the NP is then[LE3]

$$C_{\mathrm{NP}}(\tilde{s}) = 2C_\infty\sum_{m=0}^{\infty}\frac{\sinh\eta_0}{\exp[(2m+1)\eta_0]-1} = 2C_\infty(\tilde{s}^2+2\tilde{s})^{1/2}\sum_{m=0}^{\infty}\frac{1}{[1+\tilde{s}+(\tilde{s}^2+2\tilde{s})^{1/2}]^{2m+1}-1} \tag{LE8}$$

which can be summed analytically in terms of digamma functions.[LE4] The interesting property is that $C_{\mathrm{NP}}(\tilde{s}) > C_\infty$, that is, the charge separation increases as it approaches the electrode when NP and electrode are hold at constant potentials; note that NP and electrode bear charges of equal magnitudes and opposite signs. The value corresponding to a 2 nm radius NP at 1 nm separation from the electrode is $C_{\mathrm{NP}}(0.5) = 1.535C_\infty$. At this separation and shorter, the NP capacitance can be approximated by

$$C_{\mathrm{NP,NP}}(\tilde{s}) = C_{\infty}\left(\gamma + \frac{1}{2}\ln\frac{2}{\tilde{s}}\right) \quad \text{(LE9)}$$

where $\gamma = -\psi_0(1)$ is Euler's gamma.[LE5]

At the sphere surface ($\eta = \eta_0(\tilde{s})$) the space charge density is $\sigma = -\varepsilon_0\varepsilon_{\mathrm{r}}E_{\mathrm{NP},\eta}$ and the field is

$$E_{\eta,\mathrm{NP}}(\tilde{s},\theta) = -\frac{\psi_{\mathrm{NP}}}{R_{\mathrm{NP}}}\frac{2^{1/2}(\cosh\eta_0 - \cos\theta)^{3/2}}{\sinh\eta_0} \times\sum_{m=0}^{\infty}\frac{P_m(\cos\theta)}{\exp[(m+1/2)\eta_0]}\left\{\frac{\sinh\eta_0}{2(\cosh\eta_0-\cos\theta)} + (m+1/2)\coth[(m+1/2)\eta_0]\right\}. \quad \text{(LE10)}$$

In the absence of electrolyte, the force between the NP and the grounded electrode is always attractive because the induced charge density on the electrode surface has opposite charge to that on the NP. Equation (LE7) clearly shows that a NP with $\psi_{\mathrm{NP}} = 0.5\,\mathrm{V}$ induces a negative charge on a grounded electrode ($\psi_{\mathrm{E}} = 0$). The surface charge density $\sigma = \varepsilon_0\varepsilon_{\mathrm{r}}E_{\mathrm{plate},z}$ on the electrode surface for $s = 1\,\mathrm{nm}$ and $R_{\mathrm{NP}} = 2\,\mathrm{nm}$ is significant over a circular region of radius a few times R_{NP}. Figure S8 shows σ as a function of the distance $\rho = a/\tan(\theta/2)$ to the origin of Cartesian coordinates (i.e. the point of closest approach to the NP). When compared to the surface charge density on Figure S5 for $\psi_{\mathrm{NP}} = 0.5\,\mathrm{V}$ and $\psi_{\mathrm{E}} = 0$, we observe that the charge density on the electrode is around half in the presence of electrolyte, as it should be expected due to the screening of the interaction, but the spatial extension of the distribution of charge on the electrode is similar in the presence and in the absence of electrolyte. Similarly, the charge density on the NP is also around half in the presence of electrolyte, but its spatial distribution is very similar with and without electrolyte.

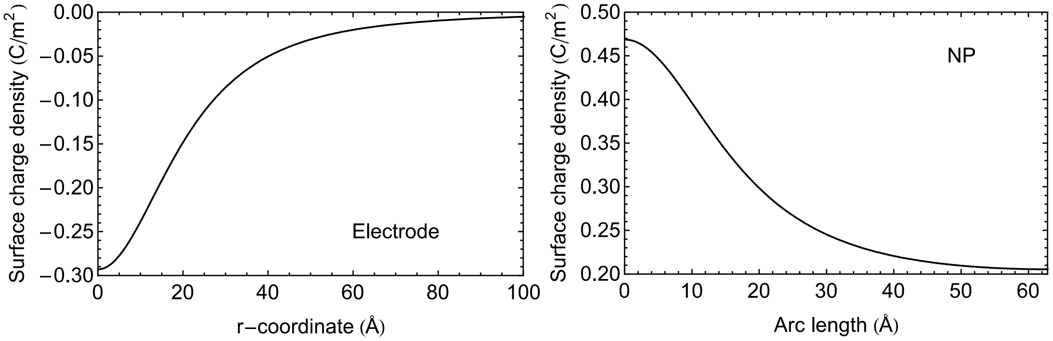

Figure S8. Surface charge densities on a grounded electrode and a 2 nm radius NP at 0.5 V separated by 1 nm in aqueous medium ($\varepsilon_{\mathrm{r}} = 78$) without electrolyte calculated from eqns (LE6) and (LE10).

7. References

JA1 G. Moy, B. Corry, S. Kuyucak and S.-H. Chung, *Biophys. J.* 2000, **78**, 2349–2363, doi: 10.1016/S0006-3495(00)76780-4.

JA2 K. H. Likharev, *Procc. IEEE* 1999, **87**, 606-632, doi: 10.1109/5.752518.

JA3 J. Cervera, J. A. Manzanares, and S. Mafe, *Nanoscale* 2010, **2**, 1033-1038, doi: 10.1039/C0NR00059K.

JA4 J. Cervera, J. A. Manzanares, and S. Mafe, *Nanotechnology* 2009, **20**, 465202, doi: doi:10.1088/0957-4484/20/46/465202.

JA5 T. Laaksonen, V. Ruiz, P. Liljeroth and B. M. Quinn, *Chem. Soc. Rev*. 2008, **37**, 1836–1846, doi: 10.1039/B713681C.

PBE1 H. Ohshima, T. W. Healy and L. R. White, *J. Colloid Interface Sci.*, 1982, **90**, 17–26, doi: 10.1016/0021-9797(82)90393-9.

PP1 D. McCormack, S. L. Carnie and D. Y. C. Chan, *J. Colloid Interface Sci.,* 1995, **169**, 177–196, doi: 10.1006/jcis.1995.1019.

PP2 S. Zhang, *Colloid J.*, 2005, **67**, 554–563, doi:10.1007/s10595-005-0133-1.

PP3 R. Hogg, W. Healy and D. W. Fuertenau, *Trans. Faraday Soc.*, 1966, **62**, 1638–1651, doi: 10.1039/TF9666201638.

PP4 W. B. Russel, D. A. Saville and W. R. Schowalter, *Colloidal Dispersions*, Cambridge University Press, Cambridge, 1989.

PP5 J. H. Masliyah and S. Bhattacharjee, *Electrokinetic and Colloid Transport Phenomena*, John Wiley & Sons, Inc., Hoboken, NJ, USA, 2006.

PP6 B. V. Derjaguin, *Discuss. Faraday Soc*., 1954, **18**, 85–98, doi: 10.1039/DF9541800085.

PP7 F. Evans and H. Wennerström, *The Colloidal Domain: Where Physics, Chemistry, Biology, and Technology Meet*, 2nd ed, Wiley-VCH, New York, 1999, sect 5.1.5.

PP8 S. Lin and M. R. Wiesner, *Langmuir*, 2010, **26**, 16638–16641, doi: 10.1021/la103046w.

PP9 J. Gregory, *J. Colloid Interface Sci.,* 1975, **51**, 44–51, doi:10.1016/0021-9797(75)90081-8.

SP1 S. Bhattacharjee and M. Elimelech, *J. Colloid Interface Sci.*, 1997, **193**, 273–285, doi: 10.1006/jcis.1997.5076.

SP2 P. Peljo, J. A. Manzanares and H. H. Girault, *Langmuir*, 2016, **32**, 5765–5775, doi: 10.1021/acs.langmuir.6b01282.

SP3 Evans, F.; Wennerström , H. The Colloidal Domain: Where Physics, Chemistry, Biology, and Technology Meet, 2nd ed, Wiley-VCH, New York, 1999, ch 5.

SP4 R. J. Phillips, Electrostatic forces between particles and planar interfaces, in Interfacial Forces and Fields. Theory and Applications, Surfactant Science Series 85, J.-P. Hsu (Ed.), Marcel Dekker, New York, ch 6, 251–188.

SP5 H. Ohshima, *J. Colloid Interface Sci.*, 1998, **198**, 42–52, doi: 10.1006/jcis.1997.5240.

SP6 S. L. Carnie, D. Y. C. Chan and J. S. Gunning, *Langmuir*, 1994, **10**, 2993–3009, doi: 10.1021/la00021a024.

SP7 M. L. Grant and D. A. saville, *J. Colloid Interface Sci.*, 1995, **171**, 35–45, doi: 10.1006/jcis.1995.1148.

SP8 J. Ståhlberg, U. Apelgren and B. Jönsson, *J. Colloid Interface Sci.*, 1995, **176**, 397–407, doi: 10.1006/jcis.1995.9952.

SP9 J. Stankovich and S. L. Carnie, *Langmuir*, 1996, **12**, 1453–1461, doi: 10.1021/la00021a024.

SP10 P. Warsynski and Z. Adamczyk, *J. Colloid Interface Sci.*, 1997, **187**, 283–295, doi: 10.1006/jcis.1996.4671.

SP11 H. Ohshima, Interaction of electrical double layers, in Electrical Phenomena at Interfaces, Surfactant Science Series 76, H. Ohshima, K. Furusawa (Eds), Marcel Dekker, New York, 1998, 57-85;

SP12 H. Ohshima, Theory of Colloid and Interfacial Electrical Phenomena, Elsevier, Amsterdam, ch 17.

LE1 W. R. Smythe, *Static and Dynamic Electricity*, 3rd ed rev, Hemisphere Publ., New York, 1989, sect. 5.082.

LE2 E. Durand, *Électrostatique Tome II Problèmes Généraux Conducteurs*, Masson, Paris, 1966, sect. 3.3.4, p. 207.

LE3 M. A. Shallal and J. A. Harrison, *Proc. IEE*, 1969, **116**, 1115–1118, doi: 10.1049/piee.1969.0209.

LE4 S. Banerjee, S. J. McCarty and E. F. Nelsen, “Exact and approximate expressions for the force between a charged conducting sphere and infinite grounded plane”, ESA 2015 C4.

LE5 J. Lekner, *J. Electrostatics*, 2011, **69**, 11–14, doi: 10.1016/j.elstat.2010.10.002.
